# Large nutritional and environmental improvements can be achieved across the product portfolios of major UK food and beverage manufacturers

**DOI:** 10.1038/s43016-026-01420-2

**Published:** 2026-09-16

**Authors:** Eleanor Hammond, Lauren Bandy, E. J. Milner-Gulland, Rachel Pechey, Mike Rayner, Peter Scarborough, Shruti Jain, Michael Clark

**Affiliations:** 1 Department of Biology, University of Oxford, Oxford, UK; 2 Nuffield Department of Primary Care Health Sciences, University of Oxford, Oxford, UK; 3 Nuffield Department of Population Health, University of Oxford, Oxford, UK; 4 School of Geography and the Environment, University of Oxford, Oxford, UK; 5 Smith School of Enterprise and the Environment, University of Oxford, Oxford, UK; 6 Oxford Martin School, University of Oxford, Oxford, UK

**Keywords:** Environmental impact, Nutrition

## Abstract

Food and beverage (F&B) manufacturers can support or hinder the transition towards a more sustainable food system. Yet, there is limited evidence about their potential to improve the nutritiousness and reduce the environmental impact of the F&B sector. Here we develop an approach to estimate the nutritional quality and environmental impact of the product lines of 44 major F&B manufacturers in the UK. We find considerable variation in terms of nutritional quality and environmental impacts across manufacturers operating within the same food retail category and within existing F&B product portfolios. We identify how changing the types of product sold by these manufacturers offers potential for nutritional and environmental benefits. For some of the F&B manufacturers assessed, changes to 10% or less of their product line could deliver more than half of the potential gains in nutritional quality or sustainability.

## Abstract (web summary)

Food and beverage manufacturers have the potential to contribute to the transition towards a more nutritious and sustainable food system. By analysing products sold by 44 major food and beverage manufacturers in the UK, this study identifies how, and to what extent, changes to product portfolios could improve the healthiness of food products while reducing their environmental impact.

## Main

The types of food that food and beverage (F&B) manufacturers produce and then sell will need to change if food systems are to transition to more nutritious and environmentally sustainable outcomes. This change can either be driven by F&B manufacturers, for instance, through voluntary product reformulation or the introduction of new more nutritious and sustainable products, or imposed on F&B manufacturers by government policy, such as by restricting promotions on less nutritious or less sustainable foods. Many F&B manufacturers have voluntarily established targets on the nutritional quality and environmental sustainability of their products and product portfolios. For example, Mars^1,2^, Nestlé^3,4^ and Tesco^5^ have targets on nutrition and pledges to reduce greenhouse gas emissions by 50% or more before 2050. However, voluntary reporting and pledges have largely failed to improve the nutritiousness and sustainability of UK food sales^6^. The UK government now plans to introduce mandatory reporting of healthy food sales for all large food companies as part of the new 10 Year Health Plan for England^7^.

The relative nutritional quality or environmental sustainability of product portfolios across different F&B manufacturers is currently unknown, as is the capacity for F&B manufacturers to support nutrition and environmental sustainability through changes within their existing product portfolios. This is because information on the nutritional and environmental impacts of food products has not been available at scale until recently (for example, see ref. ^8^). A wealth of previous research has examined the nutritional and environmental impacts and opportunities to improve food system sustainability at scales ranging from individual food products and meals^8^ to diets^9,10^, food-providing organizations^11^, countries^12^ and the global food system^13,14^, but comparatively little research exists on the nutritional and environmental impacts of F&B manufacturers.

In this study, we addressed this gap by developing and then applying an approach to quantify the nutrition and environment scores of UK F&B manufacturers. The nutrition and environmental indicators used in this analysis are directly relevant to UK policy. For nutrition, the indicator includes the nutrient profiling model that is used to establish UK food policy^15^ and that is recommended for use in mandatory reporting in the 10 Year Health Plan for England^7^. For the environment, the indicator is a composite metric that includes greenhouse gas emissions, land use, water use and eutrophication (water pollution) and has been derived following recommendations on the ecolabelling of food products in the UK^16^ and the European Union (EU)^17^. We then used this approach to identify how F&B manufacturers could improve nutritional quality and environmental sustainability by making changes within their existing product portfolios. We did this by combining information on the nutritional composition and environmental impacts per 100 g of food for >57,000 food products available in the UK market^8^ with an approach that has been used to monitor the nutritiousness of major manufacturers’ product portfolios^18–20^. This analysis builds on previous research by incorporating F&B manufacturers that account for >50% of the UK market, by assessing both nutrition and the environment, and by exploring the potential for manufacturers to improve their outcomes for nutrition and the environment. The approach developed here, if used year on year, could form the foundation of a monitoring ‘scorecard’, such as is suggested in the UK government’s 10 Year Plan for England^7^, that annually assesses how the impacts of manufacturers change through time.

## Results

### Assessment of UK F&B manufacturers’ score

We assessed the nutritional quality and environmental impacts of F&B manufacturers that collectively accounted for 51.5% of the UK F&B retail market by linking two datasets (Supplementary Fig. 1 and the Supplementary Software): (1) foodDB data from October 2019^21^, which records and derives quantitative information on the environmental impact and nutritional quality of >57,000 food products available in the UK retail market per 100 g of product^8^, and (2) a Euromonitor dataset of sales value of different categories, brands and manufacturers in the UK packaged F&B market in 2019^22^. This link between foodDB and Euromonitor allowed us to calculate the per-100-g sales-weighted nutrition and environment scores for each brand by food category combination; for instance, Mondelez UK products in the ‘Confectioneries’ food category or Tesco products in the ‘Ready Meals’ category.

We linked these datasets by first categorizing foodDB products into 1 of 31 common food product categories that were based on Euromonitor sales categories^21,22^. We did this by using the retail categories available in foodDB, which are based on the classification systems that retailers use on their online shops and which increase in specificity from Department (for example, Bakery) to Aisle (for example, Bread) to Shelf (for example, Wholemeal Bread). This provided a high-level pairing that indicated which products in foodDB belonged to each sales category. We then attributed individual products to manufacturers by searching product names and brand information in foodDB. This process resulted in the linking of 15,673 unique products in foodDB to Euromonitor, in total covering 44 manufacturers and 396 brands. Subsequently, 6 of the top 50 UK manufacturers and 1 of the top brands were removed from the analysis due to data limitations that included lack of sales data, lack of product-specific environmental and nutritional information, or because the food category was not covered by the UK nutrient profiling model (see Methods and Supplementary Fig. 1).

Once the datasets were linked, we were able to calculate the per-100-g sales-weighted environmental impacts and nutritional quality for each brand by food category. To do this, we took the mean environmental impact and nutritional quality score (see below) of all products allocated to each brand by sales category (for example, the average impact of all Tesco’s own-brand ready meals or of Haribo’s confectioneries). These manufacturer by category metrics were then weighted by sales value provided by Euromonitor to derive both category-average and manufacturer-average metrics. In total, this process provided estimates of sales-weighted nutritional and environmental impacts, capturing 88.0% of sales categories and 97.0% of sales values for the 44 manufacturers in the analysis.

In this analysis, we report nutritional quality and environmental impacts per 100 g of food product using the nutritional and environmental indicators that are used, or are being developed, for policy in the UK. We report per 100 g rather than per serving because the existing UK nutrient profiling model and current suggestions for environmental labelling on foods in the UK are derived on a per 100 g basis. For nutrition, we used the nutrient profiling model that underpins and supports UK food policy^23^. This approach includes seven characteristics of a food product to calculate an overall nutrition score ranging from −15 to 40. We linearly transformed scores to range from 0 (worst) to 100 (best), applying different transformations to foods and beverages to facilitate comparisons across these products (see Table 1 and Methods). For the environment, we combined four indicators (greenhouse gas emissions, land use, water use and eutrophication) into a single overall indicator using the approach and weightings suggested by The Institute of Grocery Distribution^16^, which is similar to the Product Environmental Footprint guidelines in Europe^17^. This composite indicator communicates the percentage contribution of 100 g of food or beverage to a person’s estimated daily allowed food-related environmental impacts to meet UK environmental policy (for example, the Courtauld Commitment to reduce greenhouse gas emissions by 50% by 2030^24^). Conversely to nutrition, therefore, low scores (for example, 0) indicate that a product is more environmentally sustainable and high scores indicate that a product is less sustainable. The environmental impacts used in this analysis account for agricultural-related impacts, but do not include impacts from processing, transportation and storage. However, the large majority of a food’s environmental impacts occur in agricultural production^25,26^. For both nutrition and environment, we converted the underlying numeric scores into an A (best, dark green) to E (worst, dark red) banding system using the thresholds suggested for nutritional and environmental labelling on food products (Table 2)^16,27^.

**Table 1:** Difference in distribution of NPM scores for F&B products

Additional multiplier applied to NPM for beverages	AD *k*-sample test statistic
1.00	1,170
1.05	1,160
1.10	1,160
1.15	1,180
1.20	1,170
1.25	1,150
1.30	1,210
1.35	1,200
1.40	1,190
1.45	1,230
1.50	1,220
1.55	1,220
1.60	1,220
1.65	1,210
1.70	1,210
1.75	1,200
1.80	1,200
1.85	1,200
1.90	1,190
1.95	1,190
2.00	1,190

The table indicates the multiplier applied to the NPM score for beverages and the Anderson–Darling (AD) *k*-sample test statistic between the distributions of the NPM scores of F&B products. The transformed NPM score for beverages was calculated as −2 × (original score) × (additional multiplier) + 70. The sample size for the Anderson–Darling test was *n* = 47,779, of which 44,164 were foods and 3,615 were beverages.

**Table 2:** Colour bands used for the environmental impact scores

Colour banding	Bands for the composite environmental impact score used in this analysis	Bands for the original IGD impact score
Green	<1.83	<5.5
Green-yellow	1.83 to <3.66	5.5 to <11
Yellow	3.66 to <7.33	11 to <22
Orange	7.33 to <11	22 to <33
Red	>11	>33

Bandings were derived from those proposed by the IGD^16^.

### Nutrition and environment scores of F&B manufacturers

**Fig. 1:**
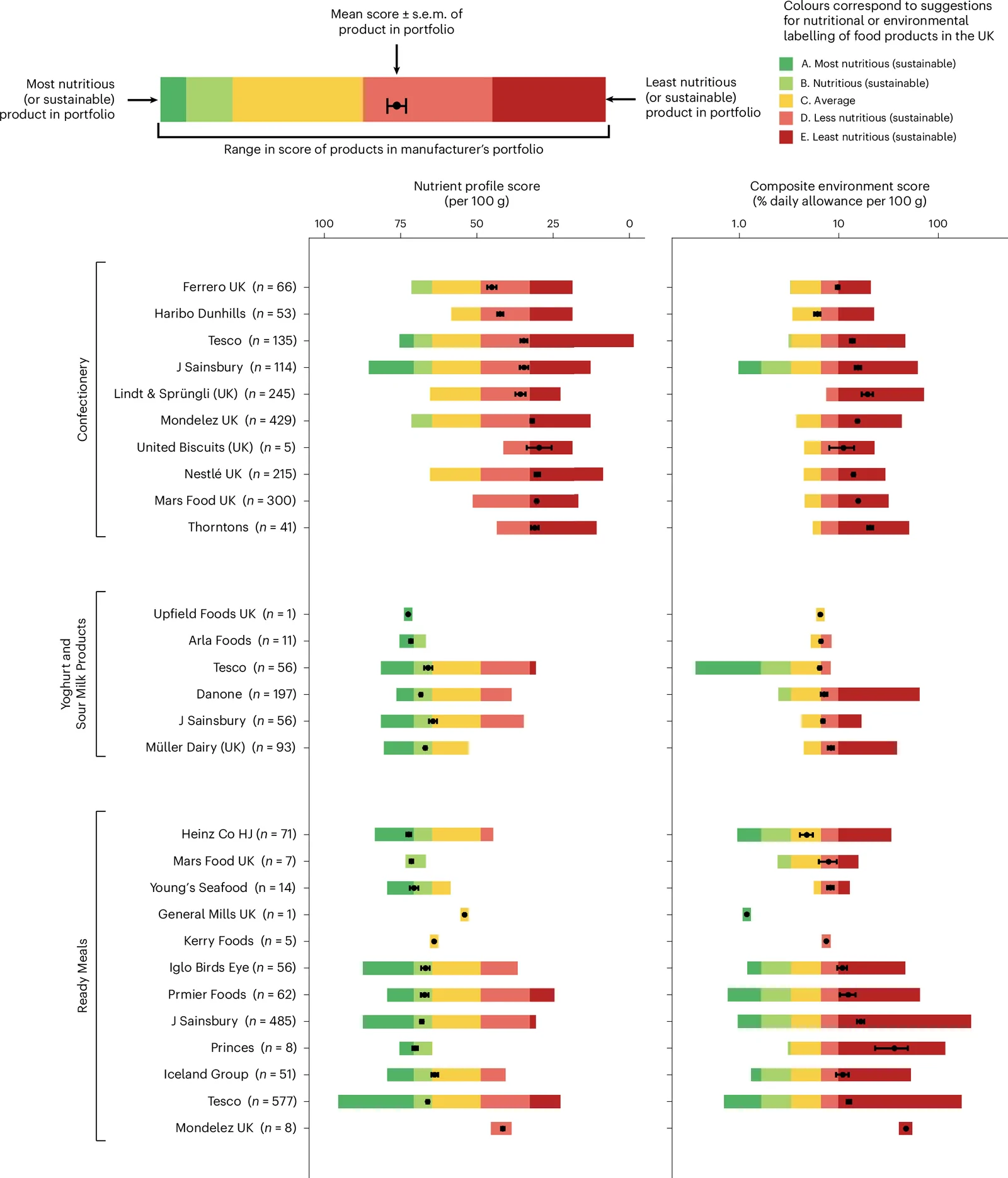
Nutrition and environment scores of F&B manufacturers in selected food categories. The coloured bars indicate the range of nutrition or environment scores in the product portfolio and range from the most sustainable or nutritious product (left side of the bar) to the least sustainable or nutritious product (right side of the bar). The colours correspond to the A (dark green, best) to E (dark red, worst) letter bandings recommended for UK food-related nutritional or environmental labelling. Black points indicate the mean score of all products for that food category and manufacturer; error bars show one standard error around the mean. The food categories and manufacturers are ordered by overall sustainability, from top to bottom. The figure is limited to the Confectionery, Yoghurt and Sour Milk Products and Ready Meals food categories. The full set of manufacturer by food category comparisons is available in Supplementary Figs. 2–4. The data used to create the figure are provided in the Supplementary Data. Source data

Across all categories, we found variation in the nutrition and environment scores of manufacturers that sell products in the same product category (Fig. 1 and Supplementary Figs. 2–4). For nutrition, there was one category (‘Rice, Pasta and Noodles’) where the average score of the best (Heinz, score A) and worst (Nestlé, score D) manufacturers varied by three letter bands, ten categories where it varied by two, thirteen categories where it varied by one, and seven categories where the best and worst manufacturer had the same average A–E score. For the environment, there were two categories (Ready Meals and ‘Sports Drinks’) where the score of the best and worst manufacturers varied by four letter bands, two categories (‘Savoury Snacks’ and Rice, Pasta and Noodles) where it varied by three bands, four categories where it varied by two, nine categories where it varied by one, and fourteen categories where the best and worst manufacturers had the same average A–E score.

We next focused on three product categories (Fig. 1), namely, Confectionery, ‘Yoghurt and Sour Milk Products’ and Ready Meals, because these categories include a variety of manufacturers, product types, and levels of variation in nutrition and environment scores both across manufacturers and within a manufacturer’s product lines. Information for other categories is available in Supplementary Figs. 2–4.

In the Confectionery category, there was smaller variation in the manufacturer’s average nutrition score than in the environment score. The worst performing manufacturer for nutrition (United Biscuits) had an average portfolio score of E, whilst the best (Ferrero) scored D, compared with the environment, where the worst (Lindt & Sprüngli) and best (Haribo Dunhills) had scores of E and C, respectively. For the environment, this difference arises because Haribo Dunhills’ products are primarily sugary gums (sugar has a low environmental impact per 100 g), compared with the mainly chocolate-based products sold by Lindt & Sprüngli (cocoa has a high environmental impact per 100 g). Within this category, the least nutritious products were consistently chocolate, toffee and fudge, the least sustainable products were chocolates with a high percentage of cocoa (for example, 95% cocoa chocolate), and the most sustainable products were jelly gums and other sugar confectionery (for example, sugary hard sweets).

The variation in scores within Yoghurt and Sour Milk Products was larger for nutrition than for the environment. The best-performing manufacturer for nutrition (Upfield Foods) had an average score of A, whilst the worst (J Sainsbury) had an average score of C. For the environment, all manufacturers had the same average score (C), which is because most products contained the same primary ingredient (dairy-based yoghurt), although the variation in environmental impacts within a manufacturer’s product line differed across manufacturers. Arla, for instance, had a small variation in environmental impacts because most of their yoghurts were either plain or with fruit. Müller in contrast had a larger variation in the environmental impacts of their products, which ranged from yoghurts with fruit (lower environmental impacts) to chocolate yoghurts (higher environmental impacts). For nutrition, the differences in this category were driven by the amount of sugar, fat (for example, non-fat or full-fat milk), salt and energy; many of the less nutritious products in the category were dessert-style yoghurts.

Within Ready Meals, there was a large variation in nutrition and environment scores, both across manufacturers and within a manufacturer’s product line. This is because most manufacturers had a wide product range that spanned from fully plant-based ready meals to mostly meat-based. Princes had one of the highest average environmental impacts because almost all of their ready meals contained meat, with their lower-impact ready meals containing chicken rather than beef. Heinz, on average, had one of the more sustainable product lines, although it varied from more sustainable (spaghetti with tomato sauce) to less sustainable (beef lasagne). For nutrition, most manufacturers had a large variation because of the diversity in their product lines. However, Princes, Young’s and Mars were exceptions to this, possibly because of their narrow product ranges (for example, Young’s only sells ready meals with fish and Mars has a pasta-based line of ready meals).

### Score profile of F&B manufacturers’ product portfolios

**Fig. 2:**
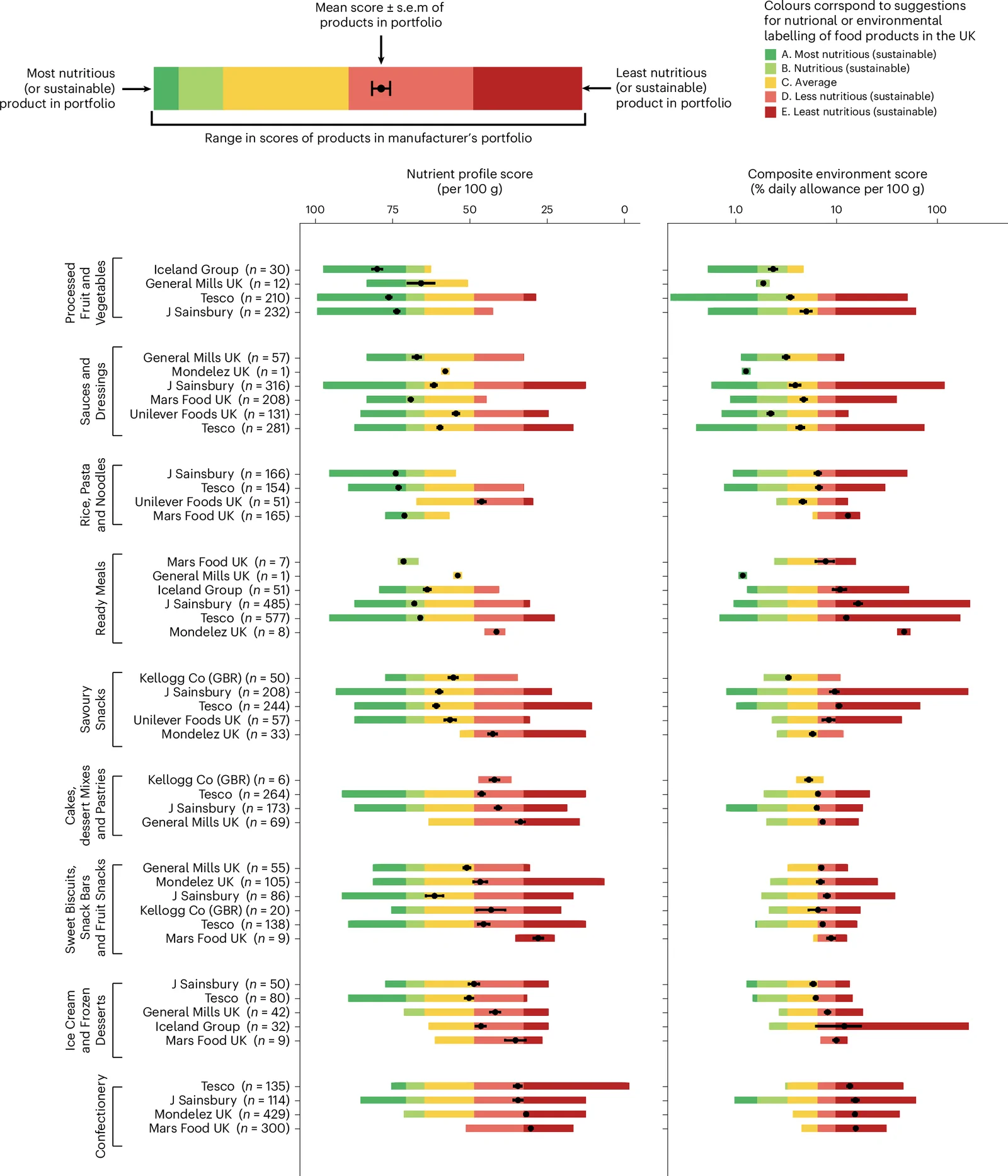
Nutrition and environment scores of F&B products of UK food manufacturers. This comparison is limited to UK retailers (Tesco, Sainsbury and Iceland) and major multinational corporations with diverse product ranges (Mars, Mondelez, General Mills, Kellogg’s and Nestlé), and to food categories where at least four of these corporations sell products. The coloured bars indicate the range of scores in the product portfolio and range from the most sustainable or nutritious (left side of the bar) to the least sustainable or nutritious product (right side of the bar). The colours correspond to the A (dark green, best) to E (dark red, worst) letter bandings recommended for UK food-related nutritional or environmental labelling. Black points indicate the mean score of all products for that food category and manufacturer; error bars show one standard error around the mean. The food categories and manufacturers are ordered by overall sustainability, from top to bottom. The full set of manufacturer by food category comparisons is available in Supplementary Figs. 2–4. The data used to create the figure are provided in the Supplementary Data. Source data

We next looked at the portfolio-level scores of corporations with broad and diverse product portfolios, including retailers such as Tesco and Sainsbury’s, and multinational corporations such as Unilever, Mars and General Mills (Fig. 2). For these manufacturers, we compared scores across their entire product lines, focusing on food categories where at least four of these corporations sell products. The scores of other manufacturers are shown in Supplementary Figs. 2–4.

Across categories and corporations, we found no consistent trend of one manufacturer selling more nutritious or sustainable products than other manufacturers (Fig. 2). When looking at the home-brand product line of retailers (for example, Tesco’s Finest), Tesco and Sainsbury’s often have fairly similar average nutrition and environment scores within a given food category, and a fairly similar variation in these scores. However, there are two exceptions to this. In Rice, Pasta and Noodles, the mean nutrition scores for Tesco and Sainsbury’s are similar, but Tesco has a larger variation in nutrition score due to a range of instant noodles that are less nutritious. In the ‘Sweet Biscuits, Snack Bars and Fruit Snacks’, Tesco has, on average, a less nutritious product line because it contains a larger proportion of sweet biscuits compared with Sainsbury’s. However, when looking at comparable products (for example, chocolate chip cookies), the nutrition score is often similar.

When looking at multinationals, such as Mars, Unilever and General Mills, we also found no trend of one manufacturer having more nutritious or sustainable product lines. For instance, Mondelez has among the least sustainable product lines in the Confectionery category, but amongst the most sustainable product lines in the Savoury Snacks and Sweet Biscuits, Snack Bars and Fruit Snacks categories. Unilever, by comparison, has one of the least nutritious but most sustainable product lines in the Rice, Pasta and Noodles category, a product line that is amongst the least nutritious but is moderately sustainable in the ‘Sauces and Dressings’ category, and a product line that is middle of the road for both nutrition and the environment in the Savoury Snacks category. Across categories, these major multinational manufacturers consistently have a large variation in both the nutrition and environment scores of their product lines.

### Potential for F&B manufacturers to improve their scores

We next investigated the potential for manufacturers to improve their nutrition and environment scores by assuming that manufacturers replaced their worst-scoring products (that is, their least nutritious and least sustainable products) in 1% increments. These products were identified based on their joint nutrition and environment score, and were replaced with products that had a median nutrition and environment score amongst all products in the same food category of the manufacturer’s existing product line. This means that Haribo would swap one gum confectionery for more sustainable and nutritious gum confectionery, whilst Thornton’s would replace a chocolate confectionery with a more sustainable and nutritious chocolate confectionery.

**Fig. 3:**
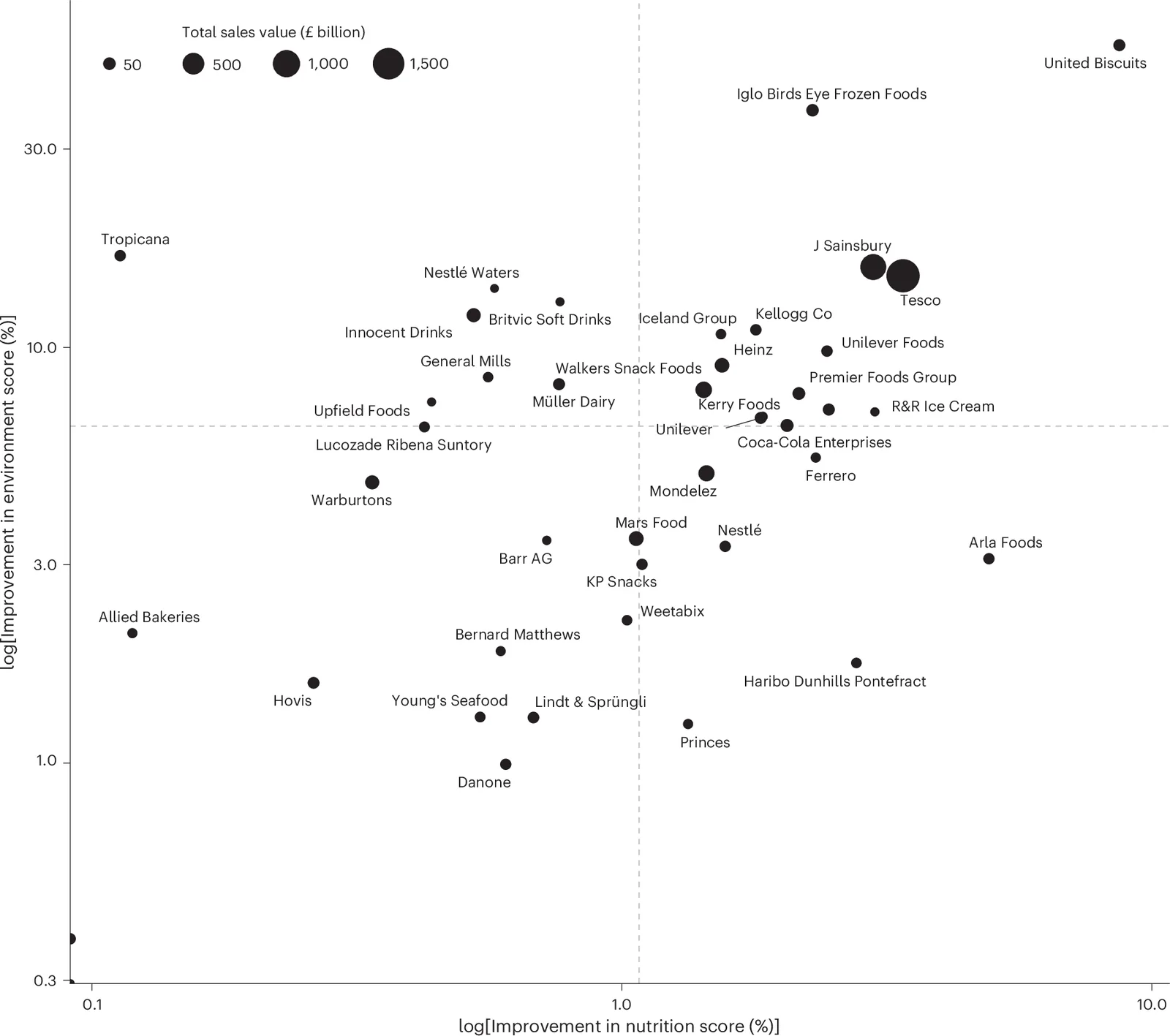
Potential improvements in nutrition and environment scores from changing 10% of an F&B manufacturer’s product line. Selected products were improved to have scores equivalent to the median nutrition or environment scores of products sold by that manufacturer in that category. Products were selected based on their joint nutrition and environment scores, their distance from median scores in the same category and the contribution of the product to the total sales value of the manufacturer. Data point size indicates the total sales value of the manufacturer and the horizontal and vertical dashed lines indicate the median potential improvement in environment or nutrition across all 44 manufacturers examined. Three manufacturers are not shown in the figure because they had no potential to improve their scores for either nutrition or environment. These are Danone Waters (0% improvement in nutrition, 51.1% improvement in environment), Red Bull (0% for both nutrition and environment) and Saputo Cheese (0% for nutrition, 0.6% for environment). The figure is plotted on log–log axes; a version of this figure with linear axes is presented in Supplementary Fig. 5. The data used to create the figure are provided in the Supplementary Data. Source data

We found varying potential for manufacturers to improve their nutrition and environment scores (Fig. 3 and Supplementary Figs. 5–7). In many cases, the manufacturers with the largest potential to improve their scores were also those with the highest sales value. For example, Tesco and Sainsbury’s, which collectively account for 28.9% of sales value in this analysis, have some of the largest scope to improve their scores for both environment and nutrition (ranked seventh and eighth for potential to improve their environment scores and fourth and sixth to improve their nutrition scores, respectively). A few other manufacturers have similar or greater potential to improve scores for one or both of the outcomes, including Iglo Birds Eye Frozen Foods, R&R Ice Cream and United Biscuits. In contrast, manufacturers with narrow product ranges often have lower scope to improve their scores, such as Hovis, Princes, Red Bull and Saputo Cheese.

When looking across manufacturers, 27 manufacturers have an estimated potential to reduce overall environmental impacts by >5%, 10 by >10% and 5 by >20%. For nutrition, 25 manufacturers have the potential to improve total nutritional outcomes by >1%, 12 by >2% and 2 by >4% (Arla and United Biscuits). These nutritional improvements translate into larger changes when looking at individual nutrients, for instance, translating into a >10% reduction in salt and sugar for six manufacturers and a >10% reduction in saturated fat for seven manufacturers. These potential benefits could be larger if manufacturers were to improve a larger proportion of their product portfolio. Potential benefits could also improve if manufacturers were to focus on improving one outcome (for example, the environment) without regards to the other outcome (for example, nutrition), although this would reduce potential benefits for the other outcome and for some manufacturers create trade-offs where, for example, improvements in the environment would lead to decreases in nutrition (and vice versa; Supplementary Figs. 5–7).

For some manufacturers, the majority of potential portfolio-level benefits are observed when changing a small proportion of their product line. For example, for 12 of the 44 manufacturers in this analysis, improving 10% or less of their product line would achieve more than half of the total potential environmental benefits if all worse-than-median scoring products were to be improved, with 9 manufacturers observing half the potential environmental benefits when 5% or less of their product line was to be improved. For nutrition, 17 manufacturers could achieve at least half of their potential improvements when improving 10% or less of their product line. There were nine manufacturers that would need to change more than one-quarter of their product line to observe at least half of the potential environmental benefits and seven manufacturers (Allied Bakeries, Iceland, Saputo Cheese, Thornton’s, Hovis, Young’s and Danone) that need to reformulate more than one-quarter of their product line to observe at least half of the potential nutritional benefits.

## Discussion

We have quantified the sales-weighted nutrition and environment scores of product portfolios of 44 major UK F&B manufacturers, accounting for 51.5% of the UK packaged F&B industry in 2019^22^. By establishing a method to assess the scores of different manufacturers in different product categories, we have identified differences within product categories and examined how changes to existing product portfolios have the potential to lead to more nutritious and environmentally sustainable outcomes. However, whilst the majority of potential nutritional and environmental benefits come from changing a small portion (<5%) of a manufacturers’ product portfolio, the potential improvements are small compared with the changes we need to meet corporate, government and societal targets on food-related nutritional and environmental outcomes. This use of the UK nutrient profiling model (NPM) and environmental metrics linked to UK policy helps to highlight the less healthy and less sustainable food products that could be targeted to reduce negative outcomes for nutrition and the environment. However, it also indicates how more fundamental changes to product portfolios and consumer behaviours will be needed, for instance, by introducing new foods that are more nutritious and sustainable, or incentivizing diet swaps towards food categories and food products that contain large amounts of fruits, vegetables, whole grain cereals, nuts and pulses—foods that are both nutritious and sustainable. This aligns with previous research, which has indicated that large-scale dietary transitions are needed to align food systems with societal targets^13,14^ and how F&B manufacturers need to be more ambitious in their contributions to food system transitions^28^.

Previous studies have focused on nutritional outcomes for F&B manufacturers^18–20,29,30^. Here we included environmental outcomes by developing an approach that pairs product-level environmental impact information with sales data from F&B manufacturers. This approach, if applied year on year, could form the foundation of a nutritional and environmental monitoring system for major F&B manufacturers, as has been recently suggested by the UK government^7^. This scorecard approach could track how the environmental impacts of F&B manufacturers have changed based on changes in product portfolios and in product sales. However, the availability of up-to-date product-level information and sales information currently limits this approach. Currently, we have to assume that products have UK-average supply chains unless the information provided on products states otherwise, that is, if the product has a stated country of origin for the full product or for specific ingredients in it. However, whilst assuming that products have UK-average supply chains is a best-guess approximation, food supply chains are consistently changing and the environmental impact of producing a given commodity often varies across countries. In addition, the environmental impact estimates used in this analysis do not include impacts associated with the processing, transportation and storage of finished food products. However, most food-related impacts stem from agricultural production, including >80% of greenhouse gas emissions and acidification and >95% of eutrophication^26^. Incorporating the environmental impacts of processing, transportation and storage would help to improve the environmental impacts estimated in this analysis, but is also unlikely to change the conclusions. In addition, the UK NPM is currently used in legislation (for example, advertising restrictions and place- and volume-based promotion restrictions) and has been subject to content, criterion and validity studies^31^. Its main limitation is that it considers only seven components in its scoring (energy, sugars, saturated fat, salt, protein, fibre, and fruit and vegetable content) and does not include measures of specific micronutrients, although these are partly captured by the composition of fruit, vegetables and protein. The model is currently used to determine whether products are in or out of scope of legislation (categorical outcome) rather than ranking all foods on a scale (continuous outcome), and further work could be done to validate the conversion method used in this study. For sales information, we further had to assume that all products in a given sales category were sold in the same quantities because the sales data were provided at the granularity of F&B manufacturer by sales category. Due to the variation in nutrition and environment scores within a sales category, this assumption could bias our results, particularly if products within the same sales category are sold in vastly different quantities. Both of these challenges could be overcome if product-level information and sales data were to be shared in a non-competitive way, as the UK’s Food Data Transparency Partnership aims to achieve^32^.

This analysis further extends previous studies by capturing a larger proportion of the UK F&B industry and by increasing granularity and specificity in the categories and brands included in the analysis. Collectively, this provides insight into which manufacturers have the largest potential to improve their nutritional and environmental performance, and how the largest manufacturers by sales value often have the largest potential to improve performance for both nutrition and environment. It also shows how many and which products they could target for change, how targeting a small proportion of an existing product portfolio (often <5%) generally brings the majority of potential benefits, and potential win-wins or trade-offs between environmental and nutritional outcomes. Further work could include analysing brands and manufacturers in the more than 50 countries where Euromonitor has data and where environmental data are available; exploring the potential benefit of across-category portfolio changes rather than the within-category changes examined here; examining how the product portfolios of F&B manufacturers would be impacted by diet changes, such as meeting food-based national dietary guidelines; exploring other aspects of sustainability covered in the United Nation’s Sustainable Development Goals^33^, such as diet affordability and acceptability; and incorporating environmental impacts associated with the post-agricultural production stages of supply chains.

This research has multiple direct policy applications. One opportunity is to align and integrate the metrics used in this framework with existing reporting and disclosure mechanisms to allow for transparent and consistent measurement and reporting across corporations and year on year. For example, these metrics could be integrated into the UK government’s Food Data Transparency Partnership^34^, an initiative that aims to improve the availability, quality and comparability of data in relation to health and environmental goals. It could also play a role in the mandatory reporting on the healthiness of F&B sales announced in the UK government’s 10-Year Health Plan For England^7^. It could also help to meet the increasing demand for quantitative metrics on nutrition and the environment, for instance, to support disclosures on environmental and social governance^35^, or comply with regulatory frameworks such as the EU’s Corporate Sustainability Reporting Directive^36^, which will require businesses that act in the EU market to disclose their environmental impacts.

All parts of the food system will need to change rapidly if we aim to create a food system that is better able to support population health and environmental sustainability^13,14^. This includes the types of F&B product that manufacturers create and then sell within the retail market. We have developed a portfolio scoring framework to benchmark the environment and nutrition scores of manufacturers, and have demonstrated its use in identifying manufacturers and products that have better or worse environment and nutrition scores, and the potential for manufacturers to contribute to a healthier and more environmentally sustainable food system by making changes within their existing product portfolios. Ultimately, however, more systemic food system transformations are needed, including ambitious shifts by manufacturers, but also shifts in the behaviours of consumers, producers, retailers, policymakers and supply chains, to create a food system that supports healthy people and a healthy planet.

## Methods

### Data types and sources

#### Food products

The information on food products was derived from foodDB^21^. foodDB is a time-stamped, weekly updated database of all food and drinks available for purchase in seven UK online supermarkets. The data collected include nutrient content, environmental impacts, ingredients, pack size, price and recommended serving size. A single snapshot of foodDB data collected in October 2019 was used for this analysis. The environmental impacts of the food products were derived using the methods from Clark et al.^8^, which used publicly available information on each product to assess their environmental impacts across five mid-point indicators: greenhouse gas emissions, land use, water use, water stress (water use scaled by regional water scarcity) and eutrophication potential. The nutrition and environmental metrics provided in foodDB are reported per 100 g of food product. In this analysis, we also report metrics per 100 g of food product for two reasons. First, serving sizes on UK food products are not regulated and can therefore be set by the manufacturer. This means that functionally similar products can have very different serving sizes, for example, serving sizes in the raw dataset for ready meals ranged from <15 g to >800 g. Second, the UK NPM used in this analysis was designed to work on a per-100-g basis and cannot be applied on a per-serving basis.

We updated the environmental impacts from Clark et al.^8^ so that the environmental impact estimates were indicative of UK-average supply chains, where supply chains are based on detailed trade matrices of the Food and Agriculture Organization^37^. For products where a different country of origin was provided, we instead assumed national-average supply chains based on the product’s stated country of origin. For ingredients where a country of origin was provided, we instead assumed that the ingredient was sourced from the stated country. In some cases, we could not perfectly pair information on country of origin (for products or ingredients) with the underlying life cycle assessment (LCA) database used in this analysis (Poore and Nemecek^26^), that is, the LCA database did not have environmental impact information for production for those crop by country combinations. In these cases (15.9% of the products in the analysis), we instead used global-average environmental impact information for those commodities.

In total, this dataset provided environmental impact information for over 61,016 unique food products that were available in the UK marketplace in October of 2019. See Supplementary Fig. 1 for a visual summary of the data inputs, methods and analyses used in this Article.

#### Sales value of food categories

Sales value data were sourced from market research company Euromonitor International^22^ and accessed through the Oxford University Library. These data provided information on the sales value of brands and manufacturers working in the UK packaged F&B market (for example, Tesco, Allied Bakeries and Nestlé UK) in 31 different food product categories (for example ‘Sweet Spreads’, ‘Edible Oils’ and Confectionery). For this analysis, we used sales data from 2019 and Euromonitor’s definition of a UK food manufacturer (“the legal entity that produces or distributes an individual or group of brands in the UK”). We limited the analysis to the top 50 UK manufacturers, identified based on their combined food and drinks sales in 2019, which collectively accounted for 61.9% of total sales value.

### Calculating product-level nutrition and environment scores

#### Deriving nutrition scores by applying the Food Standards Agency/Ofcom NPM

The UK Food Standards Agency (FSA)/Ofcom NPM was applied to each product’s nutritional composition data. Following FSA/Ofcom’s technical guidance^15^, this approach assesses the overall nutrition score of a food product based on seven characteristics per 100 g of product: energy, saturated fat, total sugar, sodium, fibre, protein, and fruit, vegetable and nut (FVN) content of the product. The first four characteristics (energy, saturated fat, total sugar and sodium) were given ‘A points‘, with each of these characteristics assessed on a scale of 0–10. The final three characteristics (fibre, protein and FVN) were given ‘C points’, each of which was assessed on a scale of 0–5. To derive the overall nutrition score, the C points were subtracted from the A points, arriving at a potential range of −15 (most nutritious) to 40 (least nutritious). This NPM was used to identify which food products can and cannot be advertised or promoted, and its derivatives have been used in other countries as the basis for NPMs that have been used to establish nutrition policy and nutrition labelling (for example, Nutri-Score^27^). The FVN content for each product was estimated from the ingredients list, where each ingredient was categorized into ‘fruit’, ‘nut’, ‘vegetable’ and ‘other’. The percentage composition of ingredients was identified where provided. For products where this information was not given, values were imputed based on the brand and category average, or, where this was not possible, a category average^8^.

To compare manufacturers’ entire product portfolios, the nutrient profiling score was converted to a scale of 1–100 using linear transformations. We used different transformations for beverages and foods because the distribution of their underlying FSA/Ofcom NPM score was different.

For foods, we used the linear expression −2 × (original score) + 70, as used by Bandy et al.^19^, where a higher score indicates a healthier product.

For beverages, we used an additional multiplication factor that minimized the difference in the distributions of the NPM scores for foods and beverages. For this, we used the Anderson–Darling *k*-sample test, which can be used to identify the similarity in two distributions. We specifically used Version 2 of the test statistic, which is more robust when testing distributions with different sample sizes. For this, we tested multiplication factors in the range of 1–2, at intervals of 0.05, using the value with the minimum test to identify the multiplier that creates the most similar distributions. As a result of this analysis, we applied an additional multiplier of 1.25 to beverages. The results of this analysis are presented in Table 1.

For visualization, after the transformations applied above, we converted the 1–100 NPM score into a green–yellow–red colour banding that aligns with the colour bands suggested by Nutri-Score. These bandings vary for foods and beverages. The following are the bandings for foods: green (NPM score 72 or greater), green-yellow (NPM score 66 to <72), yellow (NPM score 50 to <66), orange (NPM score 34 to <50) and red (NPM score <34). The following are the bandings for drinks: green (bottled water), green-yellow (NPM score 65 or greater), yellow (NPM score 55 to <65), orange (NPM score 45 to <55) and red (NPM score <45).

#### Deriving an overall composite environmental impact score

We calculated a single overall environmental impact score per 100 g of product following the recommendations developed in the Product Environmental Footprint (PEF)^17^ guidelines in Europe and The Institute of Grocery Distribution (IGD)^16^ in the UK. Both approaches provide guidelines on how to weight multiple environmental indicators into a single composite metric. The weightings for each indicator were determined based on expert opinion and were designed to align with existing EU (for PEF) or UK (for IGD) environmental guidelines.

For this analysis, we used the approach suggested by IGD^16^ because the IGD weightings are benchmarked against UK environmental policy and the focus of our analysis was the UK F&B industry. Following the IGD guidelines, we derived an overall composite environmental impact score per 100 g of each product following two steps.

The first step was to calculate the contribution per 100 g of each product to a person’s maximum daily allowed environmental impact for each of four environmental indicators (greenhouse gas emissions, land use, water use and eutrophication). This per-person daily allowance was benchmarked against existing UK environmental policy targets, with the policy targets including the Courtauld Commitment^24^ for greenhouse gas emissions (50% reduction in UK greenhouse gas emissions by 2030; 3.47 kgCO_2_-equivalent greenhouse gases per person per day, as measured using Global Warming Potential 100); no additional land use (16.3 m^2^ land per person per day)^38^; no additional water use (856 l per person per day)^38^; and existing targets on water pollution (15 gPO_4_-equivalents per person per day)^16^.

The second step was to weight these four indicators to create a composite overall environmental impact score. We used the weightings suggested by the IGD^16^, which were derived from the weightings suggested by PEF in Europe^17^, and are 52.2% for greenhouse gas emissions, 21.1% for water use, 19.7% for land use and 6.9% for eutrophication.

For visualization, we converted the overall environmental impact score into a five-tiered green–amber–red banding system. We did this by making two assumptions. First, that if a person were to consume only foods with a ‘yellow’ colour banding, their total daily food-related environmental impacts would align with UK environmental targets. Second, that the average UK adult consumes 2,000 g of food and non-alcoholic beverages per day, which is slightly below the average for UK adults when excluding alcoholic beverages^39^. We aligned the ‘yellow’ colour banding with UK environmental targets because this is similar philosophically to the UK NPM, that is, where ‘yellow’ corresponds to a food that is neither healthy nor unhealthy.

Following the above, we divided the original IGD^16^ bandings by 3, creating new bandings for the environment score used in this analysis (Table 2). We did this because the bandings suggested by the IGD for ‘yellow’ products originally had an environmental impact score in the range of 11–22. We therefore divided the suggested IGD bands by 3, such that ‘yellow’ products had a banding in the range of 3.66–7.33, and that included 5 (for example, 5% of estimated daily allowed impact to meet environmental targets per 100 g of food), which, when scaled to 2,000 g of food, would equate to aligning with the UK environmental targets described above. The bandings used in this analysis are shown in Table 2, as are the original bandings suggested by the IGD.

### Integrating datasets and allocating food products into food categories

#### Deriving common categories across foodDB and Euromonitor

The F&B product database used in this analysis (foodDB^21^) and the sales database (Euromonitor^22^) had different food categories. We therefore derived a common categorization system that provides a high-level link between these databases. This common categorization system contains 31 shared categories, which are based on Euromonitor’s categories: Edible Oils; Yoghurt and Sour Milk Products; ‘Cakes, Dessert Mixes and Pastries’; ‘Carbonates’; Ready Meals; ‘Ice Cream and Frozen Desserts’; ‘Breakfast Cereals’; ‘Concentrates’; Sauces and Dressings; ‘Other Dairy’; ‘Processed Fruit and Vegetables’; ‘Juice’; ‘Soup’; ‘Chilled and Shelf Stable Desserts’; ‘Processed Meat’; ‘Ready To Drink (RTD) Coffee’; Sweet Spreads; Confectionery; ‘Processed Seafood’; ‘RTD Tea’; ‘Butter and Spreads’; Savoury Snacks; ‘Meat Substitutes’; ‘Energy Drinks’; ‘Cheese’; Sweet Biscuits, Snack Bars and Fruit Snacks; Rice, Pasta and Noodles; Sports Drinks; ‘Drinking Milk Products’; ‘Bread’; and ‘Bottled Water’.

#### Classifying food products into Euromonitor food categories

We first developed a high-level link between the products in foodDB^21^ and the 31 food categories in Euromonitor^22^. To do this, we used the multi-tiered retailer categorization system available in foodDB, which increases in specificity from Department (for example, Bakery) to Aisle (for example, Bread) to Shelf (for example, Wholemeal Bread). This multi-tiered categorization system was provided by food retailers in their online shopping experience and was saved by foodDB when it collected data from the retailers’ websites. We used search terms on the retailer Aisle and Shelf provided for each product (see Supplementary Software for the search terms), followed by additional search terms on product names. Euromonitor product categories were categorized using a series of search terms on the product subcategory name.

Several food product types were excluded because they were not covered by the UK FSA/Ofcom NPM used in this analysis^15^. This included non-food products such as toothpaste; alcoholic drinks and low-alcohol alternatives; bubble and chewing gum; and milk formulas and other foods for babies and infants. Other product types were excluded because of inconsistent data on nutrition composition, for example, where information was variably given as solid (for example, a bouillon cube) or as diluted (for example, broth made from bouillon cubes).

In this step of product categorization, we removed a total of 18,455 products, resulting in a total of 42,561 products.

#### Pairing food products with the top 50 UK manufacturers

We next paired the remaining 42,561 products with sales value data for the top 50 UK manufacturers based on brand name and the common categories derived above. In total, this linked 15,673 F&B products from foodDB to Euromonitor categories included in this analysis, capturing 44 of the top 50 UK manufacturers and 396 brands. With this linkage, we captured 88.0% of sales categories and 97.0% of the sales value for these manufacturers.

Of the 42,561 products, 18,296 unique food products were identified as being manufactured by the top 50 manufacturers, as identified by product name and brand name. In this step, a total of 24,265 products were removed from the analysis, either because they were not manufactured by one of the top 50 UK F&B manufacturers (20,407 products) or because they were sold by one of the top 50 manufacturers but were in a product category that was not captured by the Euromonitor database (3,858 products). Euromonitor reports sales value data only when sales for that product category and manufacturer exceed a certain threshold^22^.

#### Identifying and removing products categorized into multiple Euromonitor food categories

Of the 18,296 food products linked from foodDB to one of the top 50 UK F&B manufacturers, 1,620 products were paired with multiple Euromonitor sales categories. This multicategory pairing typically resulted when foodDB products were allocated into multiple overlapping retailer categories. For example, ‘Walkers Ready Salted Crisps’ is allocated into six Shelves by one retailer: ‘Meal Deal’, ‘Fruit, Snacks and Treats’, ‘Special Offers’, ‘Single Pack Crisps’, ‘Walkers’ and ‘Food Cupboard Essentials’. This resulted in 297 additional products being removed from the analysis, but it did not cause any brand by food category combinations to be dropped from the analysis.

Because Euromonitor food categories were designed to be distinct and non-overlapping, we designed the following three-step protocol to recategorize foodDB products into a single product category where possible, or to remove products where this was not possible:

First, where possible, we applied a more specific search term to the product name to assign products to additional food categories.

Second, if the brand selling that product was already present in the newly assigned food category, we assigned the food product to that newly assigned category.

Third, if the brand was not present in the newly assigned food category, we excluded the product from the analysis.

For example, the product ‘Müller Corner Cheesecake Lime Yogurt 4 × 100 g’ was placed into the Shelf ‘All Yoghurts’ and the Shelf ‘Indulgent Yoghurts & Dairy Desserts’. This product therefore was classified into both the Euromonitor categories Yoghurt and Sour Milk Products and Chilled and Shelf Stable Desserts. After using specific search terms on the product name, this product was assigned to Yoghurt and Sour Milk Products, with the tag for Chilled and Shelf Stable Desserts removed.

In total, after this protocol, 297 unique products remained allocated into more than one Euromonitor category^22^. These products were therefore removed from the analysis. This did not result in any brand–category combinations being dropped from the analysis.

#### Identifying and removing manufacturers and brands with low sales value or category coverage

Euromonitor provides sales information for manufacturers (for example, Tesco) or brands (for example, Tesco Organic) within a category if their sales value surpasses a certain threshold^22^. This means that Euromonitor does not provide information on the sales values of manufacturers or brands with small market shares.

We checked how low sales value potentially affected the coverage of brands and manufacturers in the Euromonitor database. In this way, we identified that the manufacturers Waitrose and Morrison’s and the brand Tesco Organic (manufacturer Tesco) had low category coverage: Waitrose had products in foodDB that were allocated into 31 Euromonitor categories but sales data were present for 6 of 31 categories; Morrison’s had foodDB products in 26 categories but sales data for 14; and Tesco Organic had products in 12 categories but sales data for 1. On average, across all other brands, Euromonitor sales value data were available for 88.0% of the food product categories for which there were foodDB products: we identified 532 brand–food category combinations in the foodDB dataset, and 468 of these combinations also had Euromonitor sales value data^21,22^.

After these checks, and due to low Euromonitor category coverage compared with the products listed in foodDB^21,22^, we removed seven brands and manufacturers from the analysis: Asda, Marks and Spencer, Wrigley’s, Cow and Gate, Waitrose, Morrison’s and Tesco Organic. Asda and Marks and Spencer were removed because products from these manufacturers did not provide ingredients lists (Asda) or were not present in the F&B product database (Marks and Spencer), meaning that the sales-weighted scores of these brands could not be assessed. Wrigley’s and Cow and Gate were removed because they predominantly sell chewing/bubble gum and milk formula, respectively, which had been excluded from the analysis. Waitrose and Morrison’s and the brand Tesco Organic were removed from the analysis because they did not have a large enough market share or food product category coverage to be included in the Euromonitor sales database. This removed another 2,477 products from the analysis, resulting in the final number of 15,673 unique products, as identified by product name, included in this analysis.

### Analysis

#### Calculating sales-weighted nutrition and environment scores

After sorting foodDB products into Euromonitor food categories, we calculated average sales-weighted nutrition and environment scores per 100 g of product for each manufacturer by category combination (for example, Breakfast Cereals produced by Tesco or Confectionery produced by Mars Food UK), manufacturer (for example, Tesco and Mars Food UK) and each food product category (for example, Confectionery and Breakfast Cereals).

For each manufacturer by category combination (for example, Confectionery produced by Mars Food UK), we calculated the nutrition and environment scores of that manufacturer by category combination to be equal to the mean of all the foodDB products that sorted into that manufacturer by category combination.

To derive sales-weighted mean scores for a manufacturer’s overall product portfolio or an entire food category, we used the manufacturer by category environment and nutrition scores and the reported sales value.

For example, imagine Manufacturer A owned two brands in 2019, Brand 1 and Brand 2. Brand 1 sold products in Category X and Category Y, with reported sales in Category X of £10,000 and in Category Y of £8,000, with mean environment scores per 100 g in Category X and Category Y of 7 and 11, respectively. Brand 2 sold products in Category X and Category Z, their sales in Category X were £9,000 and in Category Z £5,000, and the mean environment scores of their products per 100 g in Category X and Category Z were 5 and 12, respectively.

In this situation, Manufacturer A’s sales-weighted mean environment score for their portfolio would be 8.22 per 100 g, calculated using the following approach:

$$\begin{array}{l}[\left(7\times 10,000\right)+\left(11\times 8,000\right)+\left(5\times 9,000\right)\\+(12\times 5,000)]/(10,000+8,000+9,000+5,000)\end{array}$$

Brand 1’s sales-weighted mean environment score would be 8.78, calculated using the following expression:

$$[\left(7\times 10,000\right)+\left(11\times 8,000\right)]/(10,000+8,000)$$

Category X’s sales-weighted mean environment score would be 6.05, calculated using the following approach:

$$[\left(7\times 10,000\right)+\left(5\times 9,000\right)]/(10,000+9,000)$$

#### Investigating options for manufacturers to improve their nutrition and environmental impacts

In this analysis, we used Sainsbury’s as a case study and assumed that the worst scoring products were improved, in increments of 1%, up to 50% of products, to have a score equivalent to the median scoring product in the same food category. This means that, in this analysis, cookies were swapped for more nutritious or sustainable cookies, rather than fresh produce.

We intended this analysis to be broadly indicative of the overall potential if a manufacturer were to change its product portfolio. We acknowledge that introducing this in real-world settings requires further consideration, for instance, the potential implications for product sales, as well as the complexities of consumer preferences for taste and texture if products were to undergo reformulation.

We identified products to improve based on their potential to improve the scores of Sainsbury’s total product portfolio using the following equation:

$$\begin{array}{l}(\mathrm{Product}\,\mathrm{score}-{\mathrm{Median}}_{\mathrm{Category}})\times {\mathrm{Sales}\,\mathrm{value}}_{\mathrm{Category}}\\\times \left(1/{\mathrm{Number}\,\mathrm{of}\,\mathrm{products}}_{\mathrm{Category}}\right)\end{array}$$

with the same equation format used to identify the potential to improve overall portfolio scores for nutrition and the environment.

Products were then ranked on their potential to improve portfolio level outcomes, with the products scoring worst for environment or nutrition (that is, least nutritious and sustainable) having the largest potential to improve outcomes. This provided two ranked scores, one for environment and one for nutrition. These two ranked scores were then summed, with the products with the lowest total (that is, combined) ranked score being those identified as having the largest potential to improve portfolio-wide outcomes.

We also conducted alternative versions of this analysis, where products were ranked based only on their potential to improve portfolio-wide environmental outcomes or only on their potential to improve portfolio-wide nutritional outcomes.

### Reporting summary

Further information on research design is available in the Nature Portfolio Reporting Summary linked to this article.

## Supplementary information

Supplementary Information Supplementary Figs. 1–7.

Reporting Summary

Supplementary Data Data used to create Supplementary Figs. 1–7.

Supplementary Software Scripts and other data inputs needed to run the analysis. An anonymized version of Euromonitor and a small subset of foodDB are provided in this folder. The full Euromonitor and foodDB datasets can not be provided due to data sharing restrictions.

## Source data

Source Data Figs. 1–3 Data from the analyses that was used to create Figs. 1–3.

## Data Availability

The Euromonitor and foodDB datasets were accessed and used under licensing agreements, which prevents them from being made publicly accessible in their original form. An anonymized version of Euromonitor and a small subset of foodDB are available as part of the Supplementary Software for the purpose of running the scripts used in this analysis. The Euromonitor data were accessed via the Bodleian Library under licence and the original dataset cannot be shared. Access to the original foodDB data can be requested via the study authors for reproducibility purposes only by emailing lauren.bandy@phc.ox.ac.uk. The data used to create all figures are provided in the Supplementary Data. Source data are provided with this paper.

## Code availability

All scripts were run using R (4.4.1). All scripts used in this analysis are available in the Supplementary Software.

## References

[1] *Climate Action Position Statement* (Mars Incorporated, 2024).

[2] *Our Mars Food and Nutrition Criteria* (Mars Incorporated, 2024).

[3] *Sustainability at Nestlé* (Nestlé, 2024).

[4] *Supporting Balanced and Sustainable Diets* (Nestlé, 2024).

[5] *Sustainability* (Tesco, 2024).

[6] Knai, C. et al. The public health responsibility deal: using a systems-level analysis to understand the lack of impact on alcohol, food, physical activity, and workplace health sub-systems. *Int. J. Environ. Res. Public Health* **15**, 2895 (2018). 30562999 10.3390/ijerph15122895 PMC6313377

[7] *Fit for the Future: 10 Year Health Plan for England* (UK Government, 2025).

[8] Clark, M. et al. Estimating the environmental impacts of 57,000 food products. *Proc. Natl Acad. Sci. USA* **119**, e2120584119 10.1073/pnas.2120584119 (2022). 35939701 PMC9388151

[9] Macdiarmid, J. I. et al. Sustainable diets for the future: can we contribute to reducing greenhouse gas emissions by eating a healthy diet? *Am. J. Clin. Nutr.* **96**, 632–639 (2012). 22854399 10.3945/ajcn.112.038729

[10] Scarborough, P. et al. Vegans, vegetarians, fish-eaters and meat-eaters in the UK show discrepant environmental impacts. *Nat. Food* **4**, 565–574 (2023). 37474804 10.1038/s43016-023-00795-w PMC10365988

[11] Taylor, I. et al. Nature-positive goals for an organization’s food consumption. *Nat. Food* **4**, 96–108 (2023). 37118582 10.1038/s43016-022-00660-2

[12] Springmann, M. et al. The healthiness and sustainability of national and global food based dietary guidelines: modelling study. *Br. Med. J.* **370**, m2322 (2020). 32669369 10.1136/bmj.m2322 PMC7362232

[13] Springmann, M. et al. Options for keeping the food system within environmental limits. *Nature*10.1038/s41586-018-0594-0 (2018). 30305731

[14] Willett, W. et al. Food in the Anthropocene: the EAT–Lancet Commission on healthy diets from sustainable food systems. *Lancet* **393**, 447–492 10.1016/S0140-6736(18)31788-4 (2019). 30660336

[15] *Nutrient Profiling Model 2004**–**2005 Technical Guidance* https://assets.publishing.service.gov.uk/media/695e87982a4a53b73d513855/NutrientProfilingModel_2004_2005_TechnicalGuidance.pdf (UK Department of Health, 2011).

[16] *Environmental Labelling for the UK Food Industry: Draft Framework Recommendation* (The Institute of Grocery Distribution, 2022).

[17] *On the Use of the Environmental Footprint Methods to Measure and Communicate the Life Cycle Environmental Performance of Products and Organisations* https://eur-lex.europa.eu/legal-content/EN/TXT/HTML/?uri=CELEX:32021H2279 (The European Commission, 2021).

[18] Bandy, L. et al. The development of a method for the global health community to assess the proportion of food and beverage companies’ sales that are derived from unhealthy foods. *Glob. Health* **19**, 94 (2023). 10.1186/s12992-023-00992-z PMC10690999 38041091

[19] Bandy, L. K. et al. Assessing the healthiness of UK food companies’ product portfolios using food sales and nutrient composition data. *PLoS ONE* **16**, e0254833 (2021). 34347807 10.1371/journal.pone.0254833 PMC8336824

[20] Martí, E., Ates, B., Wijne, M. & Gravell, M. *U.K. Product Profile 2021* (Access to Nutrition Initiative, 2021).

[21] Harrington, R. A., Adhikari, V., Rayner, M. & Scarborough, P. Nutrient composition databases in the age of big data: FoodDB, a comprehensive, real-time database infrastructure. *BMJ Open* **9**, e026652 (2019). 31253615 10.1136/bmjopen-2018-026652 PMC6609072

[22] *Euromonitor Passport: Packaged Food and Soft Drinks* (Euromonitor International, 2022).

[23] *Restricting Promotions of Products High in Fat, Sugar or Salt by Location and by Volume Price: Implementation Guidance* (UK Department of Health and Social Care, 2023).

[24] *The Courtauld Commitment 2030* (The Waste and Resources Action Plan, 2024).

[25] Crippa, M. et al. Food systems are responsible for a third of global anthropogenic GHG emissions. *Nat. Food* **2**, 198–209 (2021). 37117443 10.1038/s43016-021-00225-9

[26] Poore, J. & Nemecek, T. Reducing food’s environmental impacts through producers and consumers. *Science* **360**, 987–992 (2018). 29853680 10.1126/science.aaq0216

[27] Julia, C. & Hercberg, S. Nutri-Score: evidence of the effectiveness of the French front-of-pack nutrition label. *Ernahr. Umsch.* **64**, 181–187 (2017).

[28] Sybrig Smit, A., Fraser, E., Day, T. & Hans, F. *Corporate Climate Responsibility Monitor 2025: Food and Agriculture Sector Deep Dive* https://newclimate.org/resources/publications/corporate-climate-responsibility-monitor-2025-food-and-agriculture-sector (New Climate Institute, 2025).

[29] Vergeer, L. et al. A comparison of the nutritional quality of products offered by the top packaged food and beverage companies in Canada. *BMC Public Health* **20**, 650 10.1186/s12889-020-08828-w (2020). 32393206 PMC7216504

[30] Dunford, E. & Taylor, F. *Report on the Comparative Nutritional Profile of 1,495 Food and Beverage Products Marketed by 16 Large Companies in India* (Access to Nutrition Intitiative, 2020).

[31] Barrett, E. M. et al. Criterion validation of nutrient profiling systems: a systematic review and meta-analysis. *Am. J. Clin. Nutr.* **119**, 145–163 (2024). 37863430 10.1016/j.ajcnut.2023.10.013

[32] Food Data Transparency Partnership. *UK Government* https://www.gov.uk/government/groups/food-data-transparency-partnership (2025).

[33] *Transforming Our World: The 2030 Agenda for Sustainable Development* (United Nations, 2015).

[34] *Food Data Transparency Partnership* (UK Department of Environment and Rural Affairs and UK Department of Health and Social Care, 2024).

[35] Berg, F., Kölbel, J. F. & Rigobon, R. Aggregate confusion: the divergence of ESG ratings. *Rev. Financ.* **26**, 1315–1344 (2022).

[36] *Directive (EU) 2022/2464 of the European Parliament and of the Council of 14 December 2022, Amending Regulation (EU) No 537/2014, Directive 2004/109/EC, Directive 2006/43/EC and Directive 2013/34/EU, as Regards Corporate Sustainability Reporting* (European Commission, 2022).

[37] *FAOStat: Detailed Trade Matrix* (FAO, 2023).

[38] *Planet-Based Diets* (WWF, 2020).

[39] *National Diet and Nutrition Survey* (UK Office for Health Improvement and Disparities, 2024).

