# Supplementary material for: Large nutritional and environmental improvements can be achieved across the product portfolios of major UK food and beverage manufacturers
Source: Nat Food. 2026 Sep 16;7(9):857–68. doi: 10.1038/s43016-026-01420-2 (PMC13593567; doi:10.1038/s43016-026-01420-2)
Supplement: Supplementary file 1 [file 43016_2026_1420_MOESM1_ESM.pdf]

nature food

Supplementary information https://doi.org/10.1038/s43016-026-01420-2

Large nutritional and environmental improvements can be achieved across the product portfolios of major UK food and beverage manufacturers

In the format provided by the authors and unedited

Table of Contents:

Figures S1-S7.
Supplementary Software

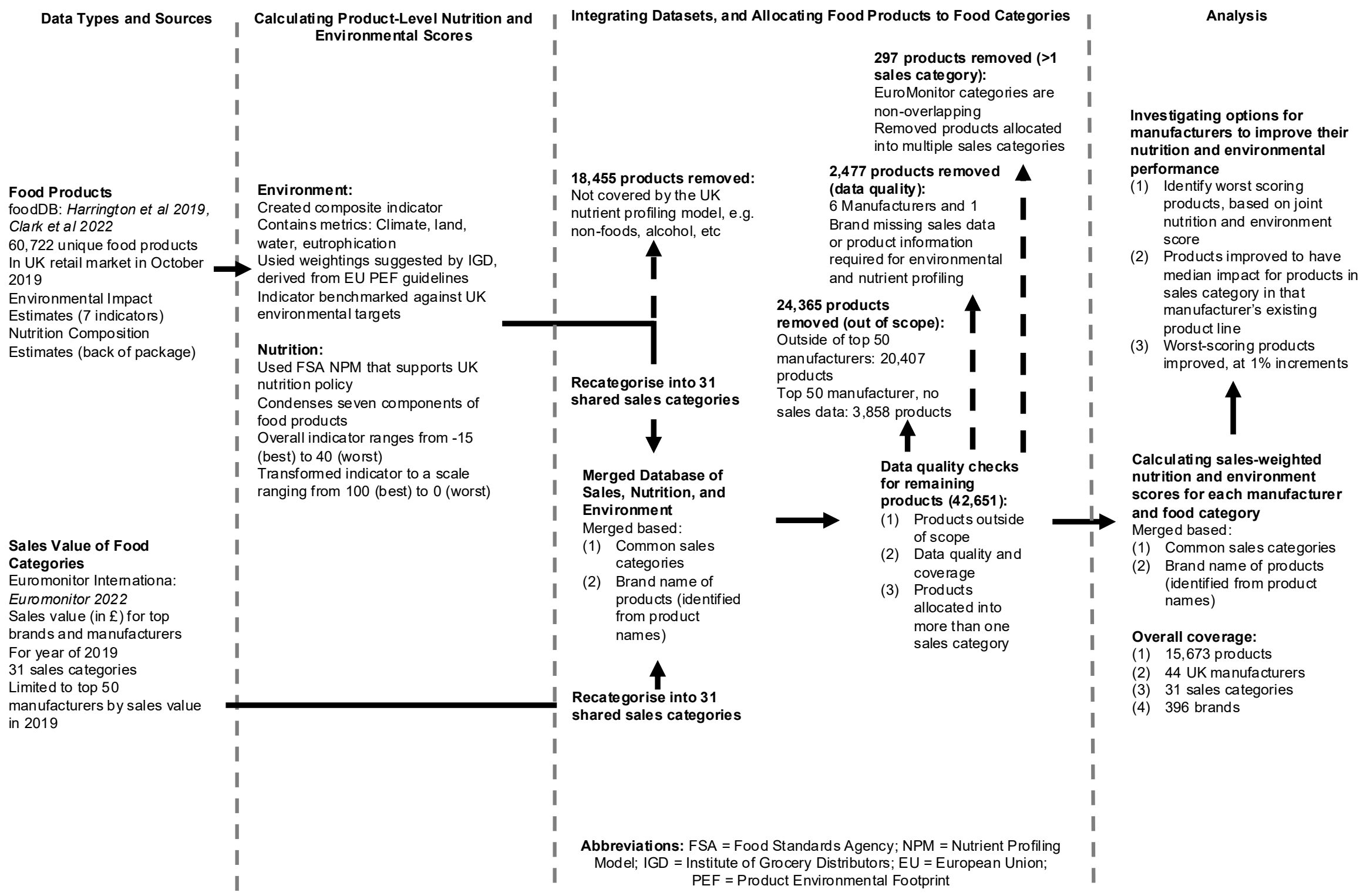

Figure S1. Flow chart of depicting data types and sources, method to calculating nutritional and environmental scores of products, approach to integrating the Euromoitor and foodDB datasets, reasons products were removed from the analysis, and analysis.

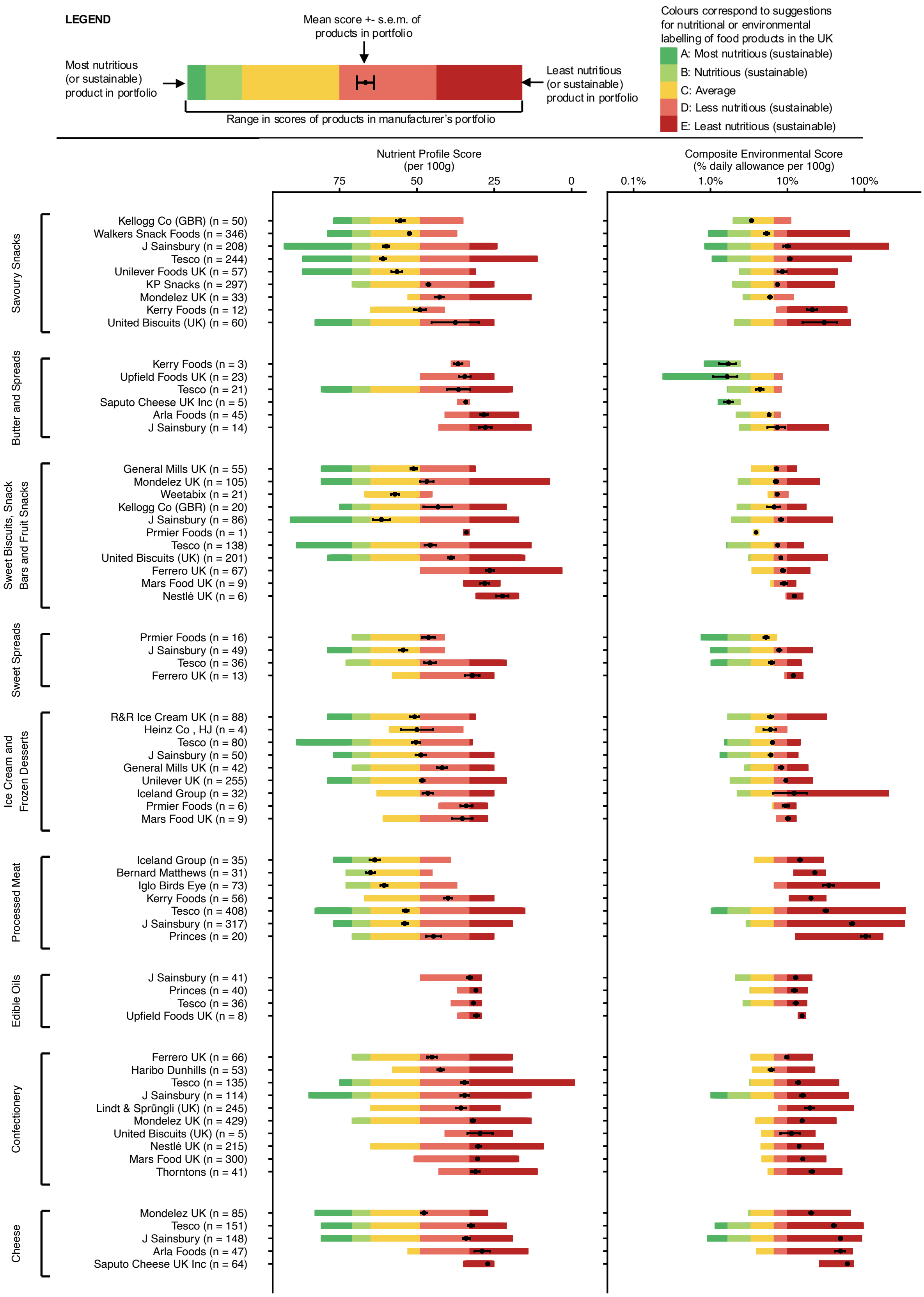

Figure S2. Nutritional and environmental scores per 100g for all manufacturer by food category combinations. Coloured bars indicate the range of scores in the product portfolio, and range from the most sustainable (nutritious; left side of the bar) to the least sustainable

(nutritious) product (right side of the bar). Colours correspond with the A (dark green; best) to E (dark red; worst) letter bandings recommendations for UK food-related nutritional or environmental labelling. Black point indicates mean score of all products for that food category and manufacturer, and error bars show one standard error around the mean. Food categories and manufacturers are ordered by overall sustainability, from top to bottom. Figure S2 shows the bottom tertile of food categories when ranked by overall nutritiousness and sustainability (i.e. least nutritious and sustainable food categories); Figures S3-4 show the other categories.

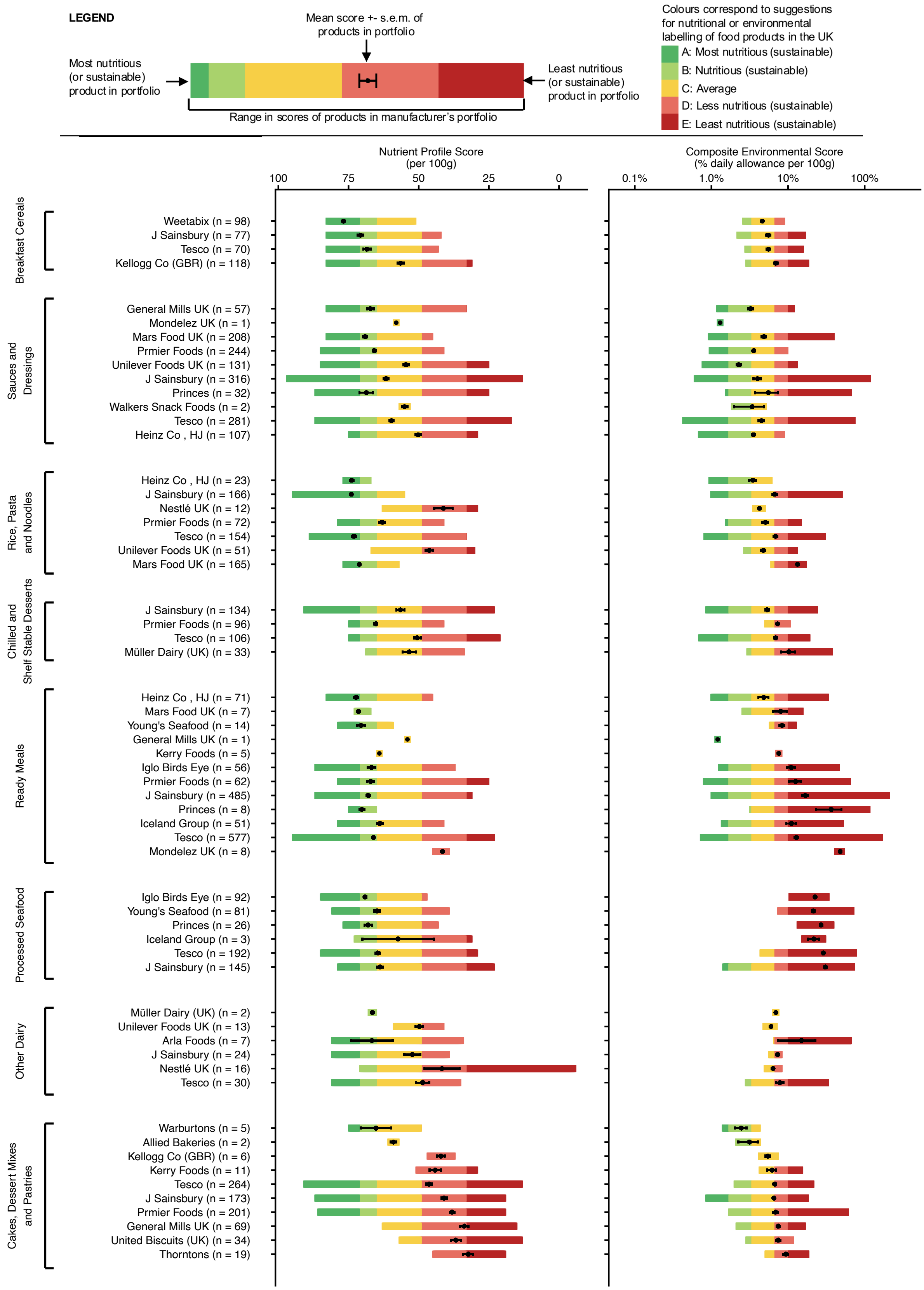

Figure S3. Nutritional and environmental scores per 100g for all manufacturer by food category combinations. Coloured bars indicate the range of scores in the product portfolio, and range from the most sustainable (nutritious; left side of the bar) to the least sustainable (nutritious) product (right side of the bar). Colours correspond with the A (dark green; best) to

E (dark red; worst) letter bandings recommendations for UK food-related nutritional or environmental labelling. Black point indicates mean score of all products for that food category and manufacturer, and error bars show one standard error around the mean. Food categories and manufacturers are ordered by overall sustainability, from top to bottom. Figure S3 shows the middle tertile of food categories when ranked by nutritiousness and sustainability; Figures S2 and S4 show the other categories.

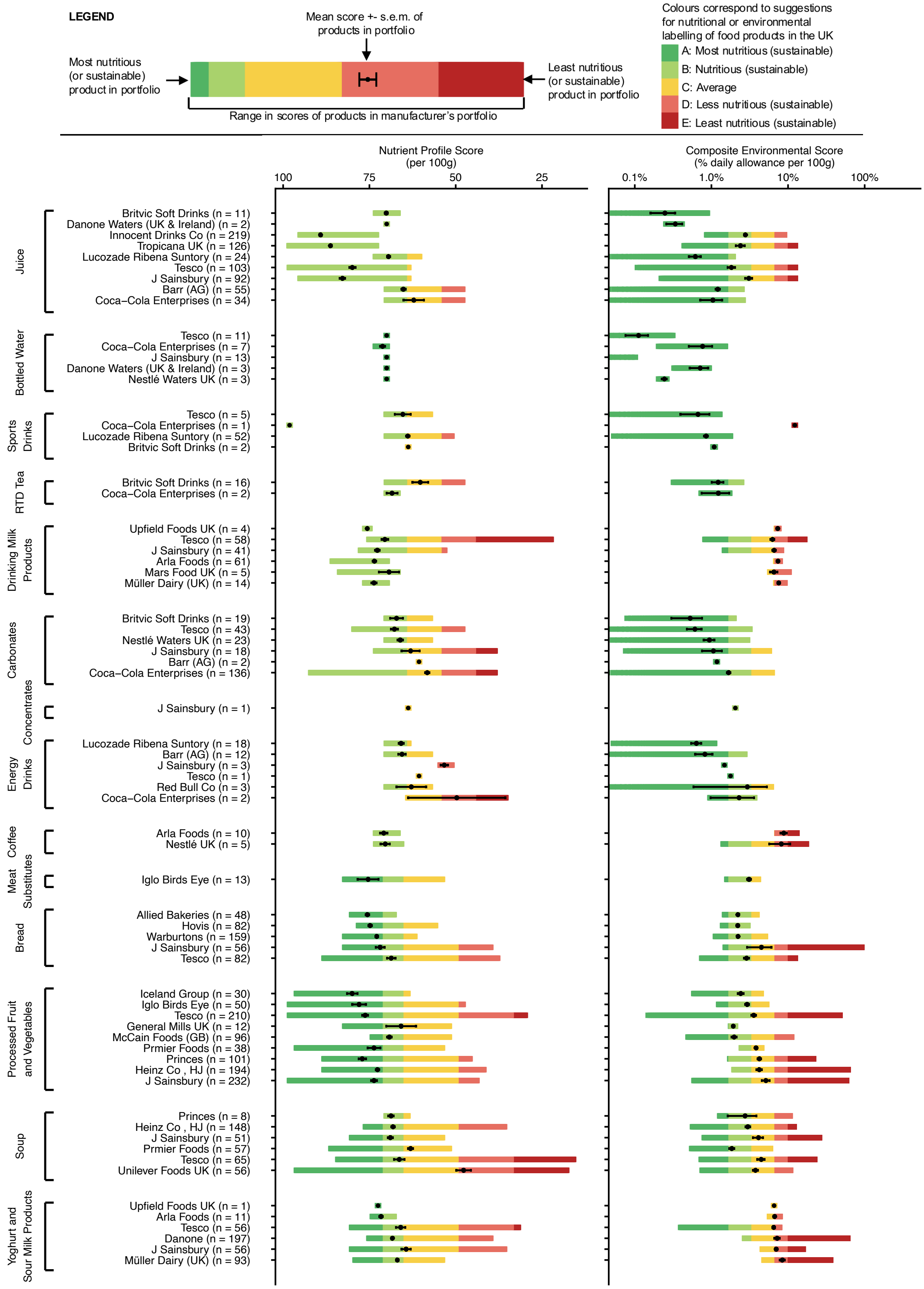

Figure S4. Nutritional and environmental scores per 100g for all manufacturer by food category combinations. Coloured bars indicate the range of scores in the product portfolio, and range from the most sustainable (nutritious; left side of the bar) to the least sustainable (nutritious) product (right side of the bar). Colours correspond with the A (dark green; best) to E (dark red; worst) letter bandings recommendations for UK food-related nutritional or

environmental labelling. Black point indicates mean score of all products for that food category and manufacturer, and error bars show one standard error around the mean. Food categories and manufacturers are ordered by overall sustainability, from top to bottom. Figure S4 shows the top tertile of food categories when ranked by overall nutritiousness and sustainability (i.e. most nutritious and sustainable food categories); Figures S2-3 show the other categories.

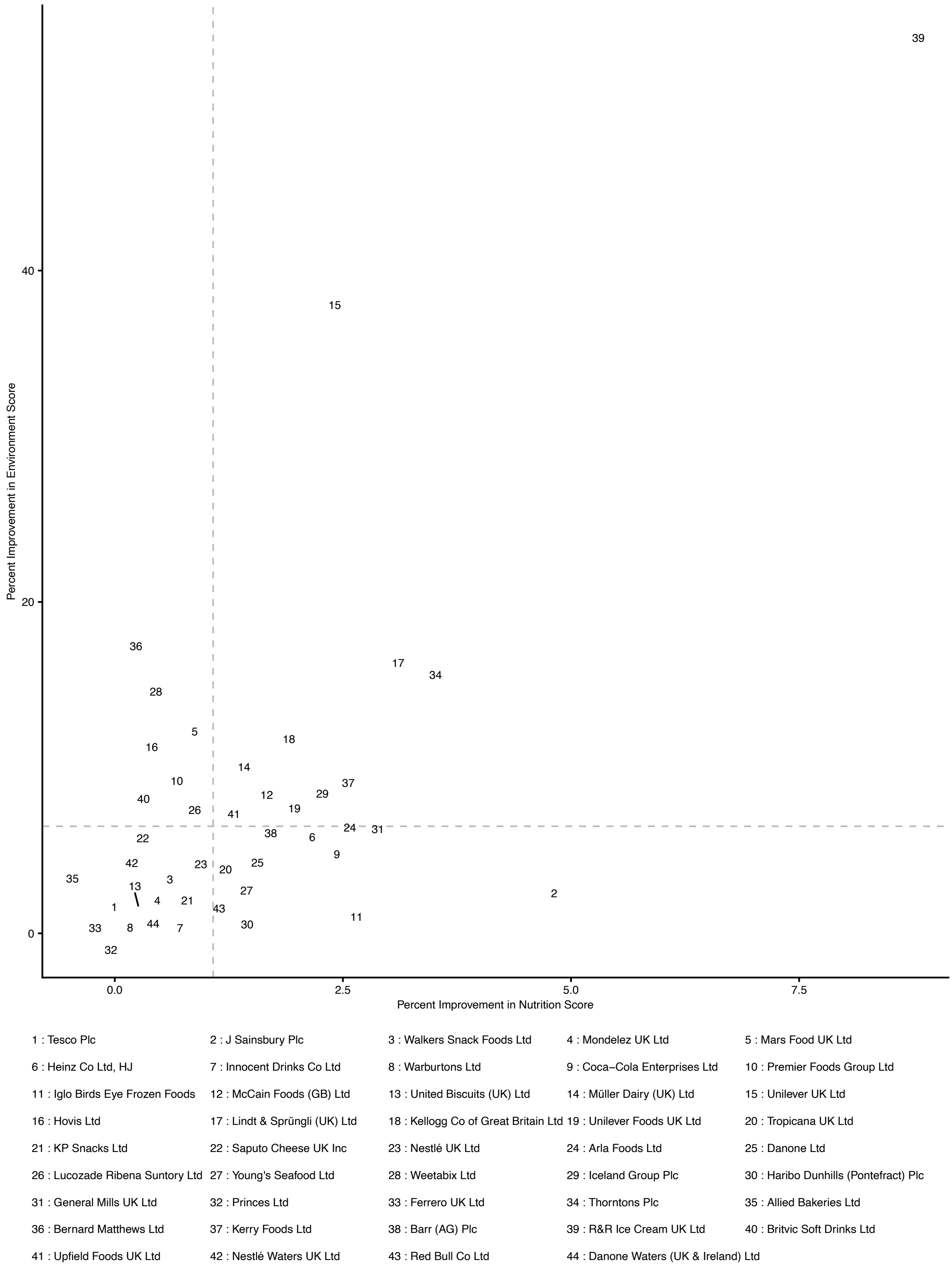

Figure S5. Potential improvements in nutritional and environmental scores if manufacturers were to improve the 10% of their products that are most harmful to nutrition or environment. Products selected were improved to have scores that are equivalent to the median nutritional or environmental score of products sold by that manufacturer in that category, and were selected based on their joint nutritional and environmental scores, their distance from median score in the same category, and the contribution of the product to total sales value of the manufacturer. Numbers correspond with manufacturers (key on bottom of figure), with

manufacturers ordered by market size (1 is largest market share; 40 is smallest market share). Horizontal and vertical dashed lines indicate the median potential improvement in environment or nutrition across all 44 manufacturers examined. Four manufacturers are not shown in the figure, because they had no potential to improve their scores on either nutritional or environment. These are Danone Waters (0% improvement in nutrition, 51.1% improvement in environment), Red Bull (0% for both nutrition and environment), Saputo Cheese (0% for nutrition, 0.6% for environment), and Tropicana (0% for nutrition, 18.1% for environment). Figure is plotted with linear axes; **Fig 3** is a version of this figure with log-log axes.

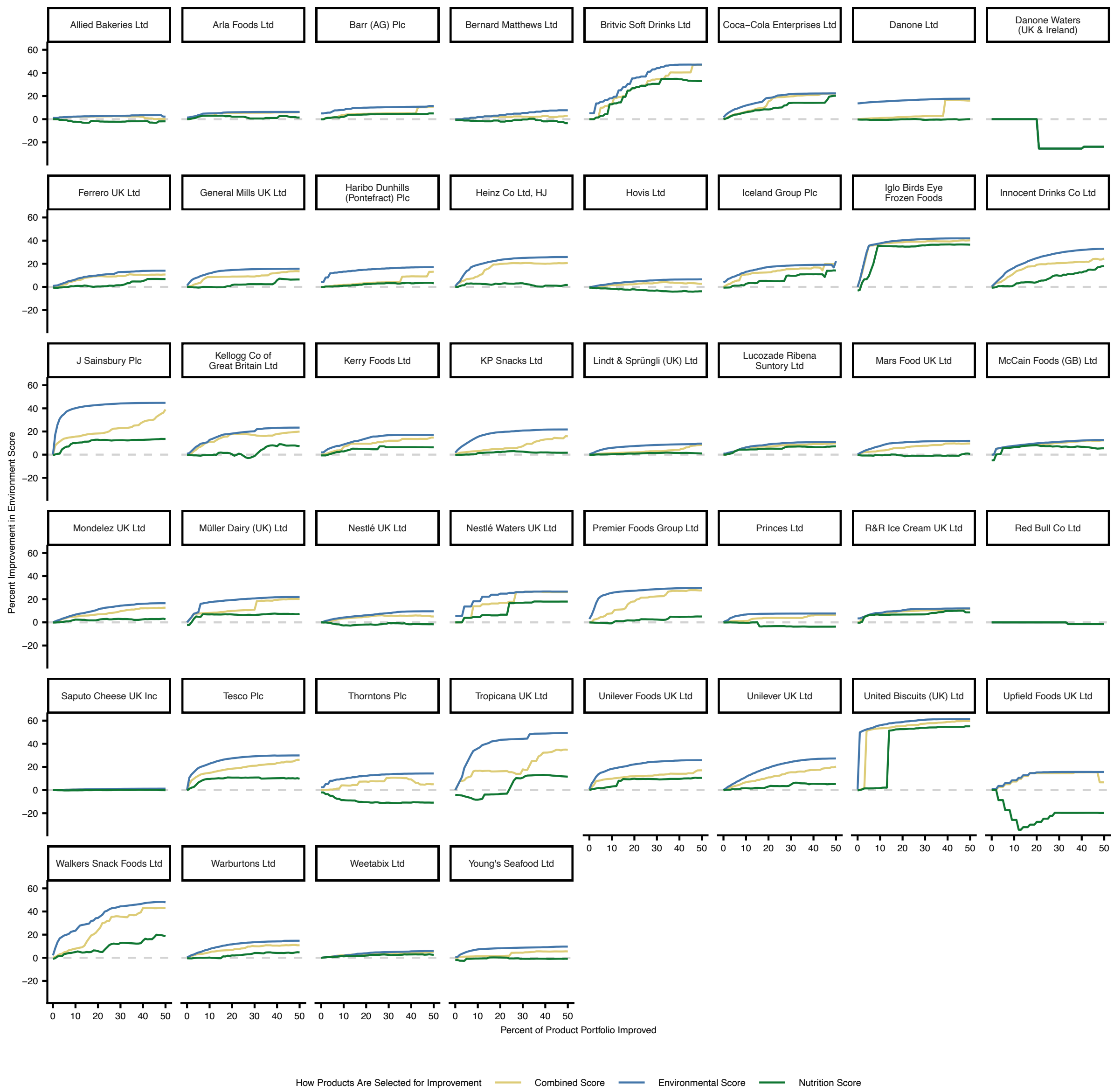

Figure S6. Estimated capacity for manufacturers to improve their environmental performance (y-axis), when improving a set percentage of their entire product portfolio (x-axis), when selecting products based on their joint nutritional and environmental score (Combined Score; yellow line), environmental impact (blue line); or nutritional impact (green line). Products selected for improvement were those with largest capacity to improve overall manufacturer-level performance, based on their score (joint, environmental, or nutritional), the distance of their score from the median scores of products in the same category, and the contribution of the product to total sales value of the manufacturer. These products were improved to have scores that are equivalent to the median nutritional or environmental score of products sold by that manufacturer in that category.

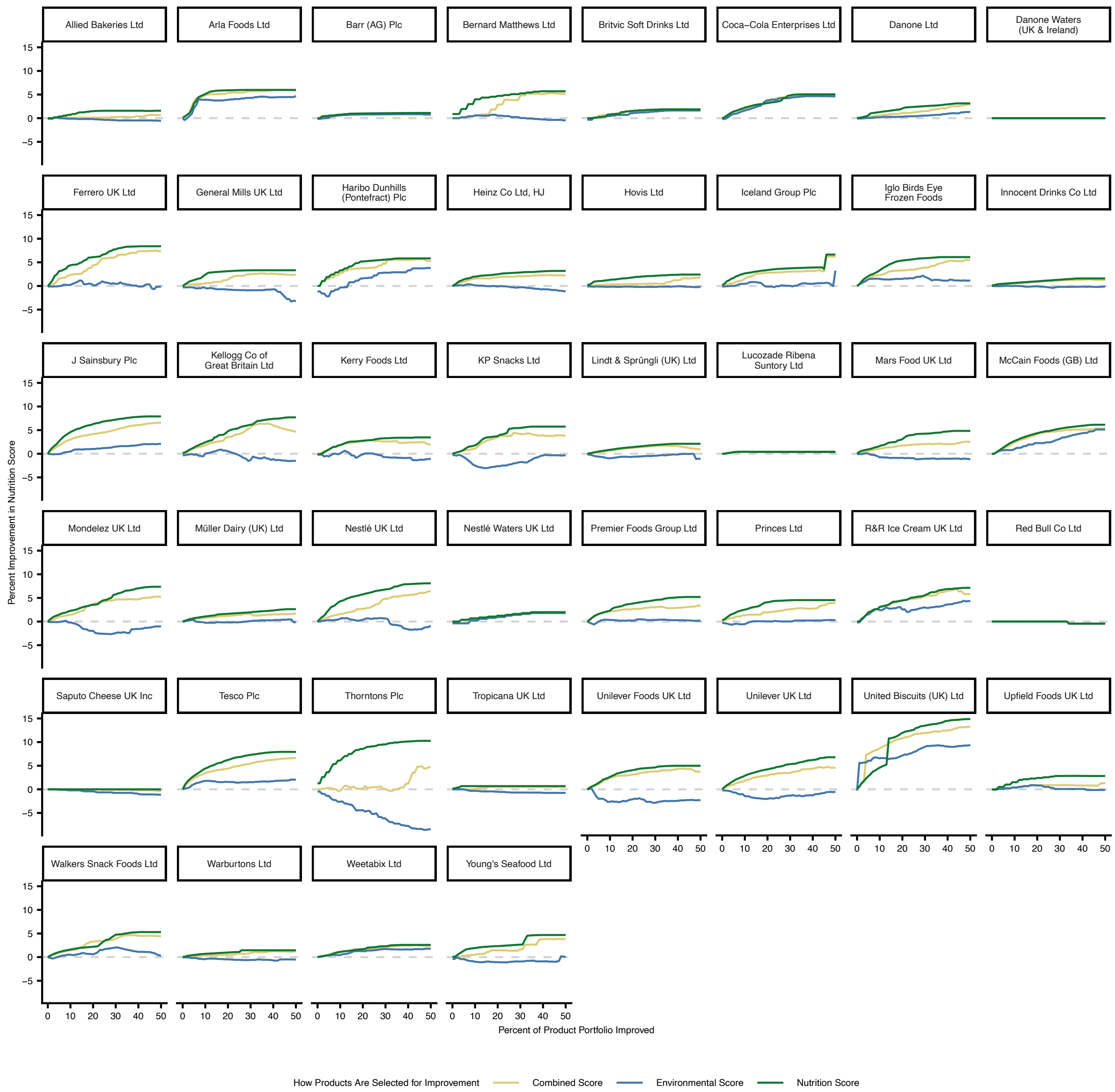

Figure S7. Estimated capacity for manufacturers to improve their nutritional performance (y-axis), when improving a set percentage of their entire product portfolio (x-axis), when selecting products based on their joint nutritional and environmental score (Combined Score; yellow line), environmental score (blue line); or nutritional score (green line). Products selected for improvement were those with largest capacity to improve overall manufacturer-level performance, based on their score (joint, environmental, or nutritional), the distance of their score from the median impacts of products in the same category, and the contribution of the product to total sales value of the manufacturer. These products were improved to have impacts that are equivalent to the median nutritional or environmental score of products sold by that manufacturer in that category.

Supplementary Software. Contains the R scripts and minimal data inputs needed to run the analysis. Full data inputs are not available due to proprietary information, but are available upon request. See also the Data Availability statement on how to request access to the data used in this analysis.
