# Supplementary material for: Large nutritional and environmental improvements can be achieved across the product portfolios of major UK food and beverage manufacturers
Source: Nat Food. 2026 Sep 16;7(9):857–68. doi: 10.1038/s43016-026-01420-2 (PMC13593567; doi:10.1038/s43016-026-01420-2)
Supplement: Supplementary file 2 [file 43016_2026_1420_MOESM2_ESM.pdf]

nature portfolio

Corresponding author(s): Michael Clark

Last updated by author(s): Jul 15, 2026

Reporting Summary

Nature Portfolio wishes to improve the reproducibility of the work that we publish. This form provides structure for consistency and transparency in reporting. For further information on Nature Portfolio policies, see our Editorial Policies and the Editorial Policy Checklist.

Statistics

For all statistical analyses, confirm that the following items are present in the figure legend, table legend, main text, or Methods section.

n/a	Confirmed	
☐	☒	The exact sample size (*n*) for each experimental group/condition, given as a discrete number and unit of measurement
☒	☐	A statement on whether measurements were taken from distinct samples or whether the same sample was measured repeatedly
☐	☒	The statistical test(s) used AND whether they are one- or two-sided<br>*Only common tests should be described solely by name; describe more complex techniques in the Methods section.*
☒	☐	A description of all covariates tested
☐	☒	A description of any assumptions or corrections, such as tests of normality and adjustment for multiple comparisons
☐	☒	A full description of the statistical parameters including central tendency (e.g. means) or other basic estimates (e.g. regression coefficient) AND variation (e.g. standard deviation) or associated estimates of uncertainty (e.g. confidence intervals)
☐	☒	For null hypothesis testing, the test statistic (e.g. *F*, *t*, *r*) with confidence intervals, effect sizes, degrees of freedom and *P* value noted<br>*Give P values as exact values whenever suitable.*
☒	☐	For Bayesian analysis, information on the choice of priors and Markov chain Monte Carlo settings
☒	☐	For hierarchical and complex designs, identification of the appropriate level for tests and full reporting of outcomes
☐	☒	Estimates of effect sizes (e.g. Cohen's *d*, Pearson's *r*), indicating how they were calculated

Our web collection on statistics for biologists contains articles on many of the points above.

Software and code

Policy information about availability of computer code

Data collection	All Scripts are provided in the Supplement as Supplemental Data 2.<br>Data inputs used were:<br>foodDB: This contains the estimated environmental impact and nutrition composition of food products available at UK retailers. A full description for this dataset is available in Clark et al 2022 (https://doi.org/10.1073/pnas.2120584119). This dataset is propriety, but is available on request with a licensing agreement via the study authors. A small subset of foodDB (10 products) is provided in Supplemental Data S2. An anonymised version of foodDB is available upon request without need for a licensing agreement.<br>Euromonitor International: This contains estimated sales revenue for different food and beverage manufacturers in different food sales categories. Access to this data was through an institutional license available for researchers at Oxford. An anonymised version of this dataset is provided in Data S2.<br>LCA data from Poore and Nemecek 2018 Science (open source, available for download as a supplement from the original paper)
Data analysis	RStudio (Version 2024.04.2+764); R (Version 4.4.1). All scripts are provided as Supplemental Data 2.

For manuscripts utilizing custom algorithms or software that are central to the research but not yet described in published literature, software must be made available to editors and reviewers. We strongly encourage code deposition in a community repository (e.g. GitHub). See the Nature Portfolio guidelines for submitting code & software for further information.

Data

Policy information about availability of data

All manuscripts must include a data availability statement. This statement should provide the following information, where applicable:

- Accession codes, unique identifiers, or web links for publicly available datasets
- A description of any restrictions on data availability
- For clinical datasets or third party data, please ensure that the statement adheres to our policy

The data used in this study are not publicly available due to privacy and proprietary reasons. Access to foodDB data is only possible through a licensed agreement restricting use of data for non-commercial uses, which can be arranged through contact with the study authors. Euromonitor data used in this analysis cannot be shared by members of the author group, but is available on Euromonitor via institutional access.

Research involving human participants, their data, or biological material

Policy information about studies with human participants or human data. See also policy information about sex, gender (identity/presentation), and sexual orientation and race, ethnicity and racism.

Reporting on sex and gender

Use the terms sex (biological attribute) and gender (shaped by social and cultural circumstances) carefully in order to avoid confusing both terms. Indicate if findings apply to only one sex or gender; describe whether sex and gender were considered in study design; whether sex and/or gender was determined based on self-reporting or assigned and methods used.
Provide in the source data disaggregated sex and gender data, where this information has been collected, and if consent has been obtained for sharing of individual-level data; provide overall numbers in this Reporting Summary. Please state if this information has not been collected.
Report sex- and gender-based analyses where performed, justify reasons for lack of sex- and gender-based analysis.

Reporting on race, ethnicity, or other socially relevant groupings

Please specify the socially constructed or socially relevant categorization variable(s) used in your manuscript and explain why they were used. Please note that such variables should not be used as proxies for other socially constructed/relevant variables (for example, race or ethnicity should not be used as a proxy for socioeconomic status).
Provide clear definitions of the relevant terms used, how they were provided (by the participants/respondents, the researchers, or third parties), and the method(s) used to classify people into the different categories (e.g. self-report, census or administrative data, social media data, etc.)
Please provide details about how you controlled for confounding variables in your analyses.

Population characteristics

Describe the covariate-relevant population characteristics of the human research participants (e.g. age, genotypic information, past and current diagnosis and treatment categories). If you filled out the behavioural & social sciences study design questions and have nothing to add here, write "See above."

Recruitment

Describe how participants were recruited. Outline any potential self-selection bias or other biases that may be present and how these are likely to impact results.

Ethics oversight

Identify the organization(s) that approved the study protocol.

Note that full information on the approval of the study protocol must also be provided in the manuscript.

Field-specific reporting

Please select the one below that is the best fit for your research. If you are not sure, read the appropriate sections before making your selection.

☐ Life sciences ☐ Behavioural & social sciences ☒ Ecological, evolutionary & environmental sciences

For a reference copy of the document with all sections, see nature.com/documents/nr-reporting-summary-flat.pdf

Ecological, evolutionary & environmental sciences study design

All studies must disclose on these points even when the disclosure is negative.

Study description

The study focuses on assessing the environmental impacts of major food and beverage manufacturers in the UK market. It assesses the performance of their product portfolio, for 44 manufacturers, on environmental and nutrition outcomes.

Research sample

The research sample is:
(a) sales of food and beverage manufacturers in the UK (from Euromonitor)
(b) data on the estimated environmental and nutrition impacts of the products sold by the food and beverage manufacturers (from foodDB)

Sampling strategy

There was no sampling strategy. All data points (e.g. food products) linked to one of the 44 manufacturers were used in the analysis.
Reasons for data exclusion are discussed in the methods, and include:
Manufacturer was not one of the top 50 in the UK by sales value
Euromonitor did not record sales value for the manufacturer (e.g. because of low sales value)

Products from foodDB could not be linked to one of the manufacturers in Euromonitor (e.g. product contained no brand information, etc)
Products (from foodDB) and sales value (from Euromonitor) were in a food category not included in the analysis (e.g. chewing gums, infant formula).

Data collection	N/A: No original data collection in this analysis. Data used in this analysis has been previous published, and this analysis focused on linking and aligning these datasets.
Timing and spatial scale	See above on timing of data collection.<br>The sales data used in this analysis was from fiscal year 2019 (from Euromonitor)<br>The environmental and nutrition impact estimates, per product of food, were from products available in the UK retail market in October 2019 (foodDB)
Data exclusions	Reasons for data exclusion include:<br>Manufacturer was not one of the top 50 in the UK by sales value<br>Euromonitor did not record sales value for the manufacturer (e.g. because of low sales value)<br>Products from foodDB could not be linked to one of the manufacturers in Euromonitor (e.g. product contained no brand information, etc)<br>Products (from foodDB) and sales value (from Euromonitor) were in a food category not included in the analysis (e.g. chewing gums, infant formula).<br>Products were allocated into two sales categories. Euromonitor sales categories are designed to be non-overlapping products, and these products were removed to avoid duplication in the analysis.<br><br>Total number of products removed at each stage of the analysis are listed in the methods.
Reproducibility	All script required to run the analysis is available in Supplemental Data 2. foodDB data used in this analysis is available upon request from the study authors under a licensing agreement.
Randomization	Not relevant - there was no randomisation in our analysis. Food products (from foodDB) were paired to sales value (from Euromonitor) based on their manufacturer (e.g. as identified by product name, branding, etc).
Blinding	There was no blinding during data analysis.<br><br>We did not blind the data because the process to pair food products to manufacturers was iterative. The original dataset containing food products contained ~60,000 unique items, which were paired to 44 manufacturers in 31 food categories. Ensuring that products were properly paired (e.g. they were sorted into the correct manufacturer and sales category) was iterative, and included:<br>(a) deriving search terms to allocate products to manufacturers and sales categories<br>(b) manually checking the pairing, to ensure products were allocated correctly<br>(c) repeating the above, until products were allocated to the correct manufacturers and food categories.

Did the study involve field work? ☐ Yes ☒ No

Reporting for specific materials, systems and methods

We require information from authors about some types of materials, experimental systems and methods used in many studies. Here, indicate whether each material, system or method listed is relevant to your study. If you are not sure if a list item applies to your research, read the appropriate section before selecting a response.

Materials & experimental systems

n/a	Involved in the study
☒	☐ Antibodies
☒	☐ Eukaryotic cell lines
☒	☐ Palaeontology and archaeology
☒	☐ Animals and other organisms
☒	☐ Clinical data
☒	☐ Dual use research of concern
☒	☐ Plants

Methods

n/a	Involved in the study
☒	☐ ChIP-seq
☒	☐ Flow cytometry
☒	☐ MRI-based neuroimaging

Plants

Seed stocks	*Report on the source of all seed stocks or other plant material used. If applicable, state the seed stock centre and catalogue number. If plant specimens were collected from the field, describe the collection location, date and sampling procedures.*
Novel plant genotypes	*Describe the methods by which all novel plant genotypes were produced. This includes those generated by transgenic approaches, gene editing, chemical/radiation-based mutagenesis and hybridization. For transgenic lines, describe the transformation method, the number of independent lines analyzed and the generation upon which experiments were performed. For gene-edited lines, describe the editor used, the endogenous sequence targeted for editing, the targeting guide RNA sequence (if applicable) and how the editor was applied.*
Authentication	*Describe any authentication procedures for each seed stock used or novel genotype generated. Describe any experiments used to assess the effect of a mutation and, where applicable, how potential secondary effects (e.g. second site T-DNA insertions, mosiacism, off-target gene editing) were examined.*
