# Supplementary material for: Large nutritional and environmental improvements can be achieved across the product portfolios of major UK food and beverage manufacturers
Source: Nat Food. 2026 Sep 16;7(9):857–68. doi: 10.1038/s43016-026-01420-2 (PMC13593567; doi:10.1038/s43016-026-01420-2)
Supplement: Supplementary file 3 [file 43016_2026_1420_MOESM4_ESM.zip › Data_S2_Run_Folder/Read Me.docx]

**Read Me:**

This folder contains the scripts and a subset of data used for the manuscript titled: “**Assessing the Nutritional and Environmental Impacts of Major UK Food and Beverage Manufacturers”.** This file contains descriptions of these scripts and datasets, as well as information on how to run the scripts.

**Scripts:**

There are a total of 8 scripts used in this analysis:

- 1.0_initialcategorisations_2024.06.10.R
- 2.0_market_share_20240610.R
- 3.0_product_categorisation_and_matching_2024.07.11.R
- 4.0_Fig1_Brand_by_Category_2025.10.08.
- 4.0_Fig2_MainNBOs_2025.10.08.R
- 4.0_Fig3_S3_S4_Improving_performance_update_2025.10.08.R
- 4.0_FigS2_Brand_by_Category_others_2025.10.08.R
- Functions_tidy_13.09.2023.R

The scripts run in numeric order. That is, the script titled ‘1.0_...’ needs to be run before the script titled ‘2.0_…’, which needs to be run before the script titled ‘3.0_...’.

The scripts titled ‘4.0_...’ are the scripts used to create the figures used in the manuscript, and also save the data used to create the figures (this data is provided in Dataset S1). The ‘4.0_...’ scripts can be run in any order.

The script titled ‘Functions_tidy_...’ contains the functions that are called in the other scripts. This ‘Functions_tidy_...’ script does not need to be run.

All scripts in this analysis were run using R version 4.4.1.

A short description of each script is below:

*1.0_initialcategorisations_2024.06.10.R:* This script creates initial linkages between the product-level dataset used in this analysis (named foodDB) and the sales dataset used in this analysis (named EuroMonitor). This linkage is done by using string searches on product names (in foodDB), and then allocating products to food and beverage manufacturers based on identifying information in the product name (e.g. brand name).

This matching is done at two levels: on local brand names and national brand owners. There can be multiple local brand names allocated to each national brand owner. For example, the local brand names ‘Galaxy’, ‘Maltesers’, ‘Mars’, ‘Celebrations’, ‘Dolmio’, etc… are all under the national brand owner ‘Mars Food UK Ltd’.

*2.0_market_share_20240610.R:* This script calculates market share and market value by national brand owner. It aggregates the market share of local brand names into each national brand owner. It also removes national brand owners with low market share from the analysis.

*3.0_product_categorisation_and_matching_2024.07.11.R:* This script categorises each product (from foodDB) to food and beverage categories (provided by EuroMonitor). This process is done using string searches on a combination of product names (in foodDB) and supermarket ‘Aisles’ (also provided in foodDB).

This script also removes products from the analysis (e.g. those that do not correspond with any of the top 50 national brand owners, or that are not categorised into sales categories of a national brand owner). It also removes products that have been categorised into multiple sales categories. This is because the EuroMonitor sales categories are designed to be non-overlapping – i.e. a product can by definition only be allocated to a single category.

*4.0_Fig1_Brand_by_Category_2025.10.08:* This makes Fig 1, and saves the data for Fig 1.

*4.0_Fig2_MainNBOs_2025.10.08.R:* This makes Fig 2, and saves the data for Fig 2.

*4.0_Fig3_S3_S4_Improving_performance_update_2025.10.08.R*: This makes Figs 3, S3, and S4. It also saves the data for these figures.

*4.0_FigS2_Brand_by_Category_others_2025.10.08.R:* This makes Fig S2, and saves the data for Fig S2.

*Functions_tidy_13.09.2023.R:* This script contains the functions that are run in the other scripts.

**Data:**

This folder also contains a subset of the raw data needed to run the analysis. All data inputs that have no limits to sharing are provided in full. Data limits that have limits to sharing are either provided as a subset of the full dataset (foodDB), or with the structure of the full dataset but with randomised values (EuroMonitor). This is to ensure that sharing of this dataset meets data sharing limits associated with this data.

Short descriptions of the foodDB and EuroMonitor dataset are below.

*foodDB:* This dataset contains product-specific environmental and nutrition impact estimates. The full dataset contains >50,000 products. The version of the dataset provided in this folder contains 10 products due to data sharing limitations.

The environmental indicators in foodDB that are also used in this analysis include: greenhouse gas emissions (kg CO2e), water use (liters water), eutrophication (gPO4e), and land use (square meters). All impacts are reported per 100g of finished product.

The nutrition indicator in foodDB that is also used in this analysis includes: the UK Nutrient Profiling Model. This scores food products on a 0 (less nutritious) to 100 (more nutritious) scale. This score is based on seven components of a food: salt, sugar, energy, saturated fat, protein, fibre, and the proportion of the product that is a fruit, vegetable, or nut. A full description of how seven components of a food are used to calculate each food’s nutrient profile score is described elsewhere in depth (e.g. [see here](https://www.gov.uk/government/publications/the-nutrient-profiling-model)).

*EuroMonitor:* This dataset contains the sales value of each national brand owner in each food and beverage category. The version of the dataset provided in this folder contains randomised sales values due to data sharing limitations.

Food and beverage categories are provided by EuroMonitor. In this analysis we use EuroMonitor’s ‘Subcategory’

The sales value is provided as million Great British Pounds (£). National brand owners with low market share in a food category are excluded from the EuroMonitor database.

**Expected Run Time:**

All scripts in this folder should take less than 30 minutes to run on a normal desktop computer.

**Version of R and RStudio:**

All code in this analysis was run using R Version 4.4.1 and RStudio Version 2024.04.2+764.

The packages required to run the scripts are noted at the top of each script. Packages are available for download from a CRAN repository. Any additional functions required to run the scripts (i.e. those not in an R package) are provided in the script named “Functions_tidy_13.09.2023.R”.
